# Anticancer Effects of Ascorbic Acid: Not All Sides Fit All

**DOI:** 10.3390/cancers17172877

**Published:** 2025-09-01

**Authors:** Uche O. Arunsi, Jeremiah O. Olugbami, Adegboyega K. Oyelere

**Affiliations:** 1 School of Chemistry and Biochemistry, Georgia Institute of Technology, 901 Atlantic Drive, Atlanta, GA 30332, USA; 2 Parker H. Petit Institute for Bioengineering and Bioscience, Georgia Institute of Technology, 315 Ferst Drive, Atlanta, GA 30332, USA

**Keywords:** cell viability assessment, ascorbic acid, cancer cell proliferation, MTS assay, MTT assay, PI/Triton X-100 assay, cell imaging, apoptosis, ROS, lipid peroxidation

## Abstract

**Simple Summary:**

This study examines the impact of ascorbic acid (vitamin C) on the growth of cancer cells. While vitamin C is recognized for its health benefits, it can also serve as a potent agent that kills cancer cells by increasing harmful molecules known as reactive oxygen species. Traditional laboratory tests may yield misleading results when evaluating this effect; therefore, we employed a more precise method to assess ascorbic acid’s impact on various types of cancer and normal cells. We observed that ascorbic acid notably decreased the growth of cancer cells, particularly those with specific hormone receptors, while having a lesser effect on normal cells. The study also elucidates how vitamin C induces cancer cell death through various biological processes. These findings could enhance the way researchers test cancer treatments and foster the development of safer, more targeted therapies.

**Abstract:**

**Background/Objectives:** Ascorbic acid (AA)is a micronutrient with concentration-dependent anticancer properties, acting either as a reactive oxygen species (ROS) scavenger or inducer. **Methods**: Conventional redox-based assays such as MTS/MTT often overestimate cell proliferation due to AA’s interaction with tetrazolium salts, leading to increased formazan production. To overcome this limitation, we employed the Propidium Iodide Triton X-100 (PI/TX-100) assay to evaluate AA’s cytotoxic effects across a diverse panel of cancer and normal cell lines, including prostate (22Rv1, C4-2B, DU-145, LNCaP), breast (MCF-7, MDA-MB-231, MDA-MB-453), lung (A549), liver (HepG2, SK-HEP-1, Huh7), and kidney (Vero) cells. **Results**: AA significantly suppressed cancer cell viability compared to normal cells (RWPE1 and Vero), with the strongest effects observed in hormone receptor-positive lines. The relative sensitivity to AA followed distinct patterns within each cancer type. Mechanistically, AA-induced cell death involved ROS generation, lipid peroxidation, cell cycle arrest, ferroptosis, apoptosis, and downregulation of pyruvate dehydrogenase kinase 1 (PDHK1). **Conclusions:** These findings further support the potential of AA as a selective anticancer agent and highlight the importance of assay choice in evaluating its therapeutic efficacy.

## 1. Introduction

The global burden of cancers has become a significant concern. According to the 2020 Global Cancer Statistics, there were an estimated 19.3 million new cancer cases and 10 million cancer-related deaths [1]. These figures reflect a 6.67% increase in incidence and a 4.17% increase in mortality compared to the 2018 data [2]. Despite advancements in diagnostic and therapeutic approaches aimed at reducing cancer morbidity, improving survival rates, and enhancing quality of life, cancer remains the second leading cause of death globally, trailing behind cardiovascular diseases [3]. Various strategies, including surgery [4], chemotherapy [5], radiotherapy [6], and immunotherapy [7], have been employed to prevent, manage, and treat cancers. However, the quest for the development of effective therapeutics persists. One of the challenges associated with existing cancer treatments is the incidence of treatment-related side effects and adverse drug reactions. These adverse effects can include nausea, vomiting, constipation, anorexia, malabsorption, weight loss, anemia, fatigue, gastrointestinal impairment and damage to critical organs of the body [8,9]. Hence, there is an urgent need for novel, broad-acting and efficient therapeutic drugs with an excellent safety margin.

Ascorbic acid (AA) is a water-soluble vitamin that exhibits diverse biological effects. In the cell, AA demonstrates a dual redox role, acting as a reactive oxygen species (ROS) scavenger at low concentrations and as a ROS generator at pharmacological doses. This concentration-dependent behavior enables AA to modulate oxidative stress, influence redox-sensitive signaling pathways, and chelate transition metals [10,11,12,13,14]. In tumor models, AA has been shown to inhibit cancer growth both in vitro and in vivo [15,16,17]. The therapeutic interest in AA dates back to 1959 when William J. McCormick observed scurvy-like symptoms and low AA levels in cancer patients, hypothesizing that AA might protect against tumors by enhancing collagen synthesis [18]. He proposed that increased collagen production could strengthen cell adhesion and impede tumor spread.

Building on this hypothesis, L. Benade, Howard T. and Burk D. reported that AA was toxic or lethal to Ehrlich ascites carcinoma (EAC) cell line [19]. Subsequent findings by Ewan and Allan Campbell demonstrated AA’s ability to suppress hyaluronidase activity, an enzyme that degrades the extracellular matrix (ECM) and facilitates metastasis. Encouraged by these findings, Cameron and Campbell administered high-dose AA to 50 terminal cancer patients and published a case report indicating clinical benefits [20]. Cameron later collaborated with Linus Pauling, leading to the publication of two influential reviews on AA’s role in orthomolecular medicine [21,22]. Their clinical trials involving 100 terminal cancer patients and 1000 retrospective controls suggested improved quality of life and a fourfold increase in survival among AA-treated patients [23,24]. However, these findings were challenged by Creagan and Moertel in 1979, whose controlled studies found no significant therapeutic benefit of intravenous AA in advanced cancer patients [25,26]. These conflicting results have led to a reevaluation of AA’s role, with current interest focusing on its use as an adjuvant in combination therapies.

Emerging evidence supports AA’s selective cytotoxicity in cancer cells. While normal cells tolerate pharmacological doses of AA, cancer cells exhibit increased sensitivity, leading to cell death. This effect is primarily mediated by AA-induced oxidative stress, particularly through the generation of hydrogen peroxide (H_2_O_2_) during its oxidation to mono- and dehydroascorbate [27,28,29]. H_2_O_2_ accumulation disrupts mitochondrial function and reduces ATP production. Additionally, AA downregulates key metabolic and survival pathways, including mammalian target of rapamycin (mTOR), GAPDH activity, and enzymes involved in glycolysis and autophagy. These effects have been observed in MIA-PaCa-2 pancreatic cancer cells [30], prostate cancer cells (C4-2B, LAPC4, LNCaP, PC3, PC3/GFP-LC3, and 22Rv1) [31], and KRAS-mutant colorectal cancer cells [15]. AA also modulates epigenetic and hypoxic responses by regulating ten-eleven translocation (TET) enzymes, hypoxia-inducible factor 1-alpha (HIF-1α), and glucose metabolism [32,33].

In the current study, we have assessed the overall impact of AA on diverse cancer and normal cells, including prostate cancer cells (22Rv1, DU-145, C4-2B, and LNCaP), breast cancer cells (MCF-7, MDA-MB-231, and MDA-MB-453), liver cancer cells (HepG2, Huh7, and SK-HEP-1), non-small cell lung cancer cell (NSCLC) (A549), and normal cells (RWPE1 and Vero), to obtain insight about drivers of cancer cell responsiveness to AA. Our findings reveal that cancer cells with high expression of nuclear hormone receptors, such as androgen and estrogen receptors, are more predisposed to AA-mediated tumor cell death.

## 2. Materials and Methods

### 2.1. Cell Culture and Cell Viability Assays

The human breast (MCF-7, MDA-MB-231, and MDA-MB453), prostate (LNCaP and DU-145), liver (HepG2, Huh-7, and SK-HEP-1), and lung (A549) cancer cell lines, along with normal cell lines (Vero and RWPE1), were obtained from American Type Culture Collection (ATCC). Cell lines were verified by ATCC and only used while passage numbers were low (<20). MDA-MB-453, LNCaP, C4-2B, and 22Rv1 cell lines were routinely cultured in phenol-free RPMI (Invitrogen, Carlsbad, CA, USA) with fetal bovine serum (10%), L-Glutamine (1%), and penicillin–streptomycin (1%); DU-145, SK-HEP-1, and HepG2 cell lines were maintained in phenol-free MEM (Invitrogen) with fetal bovine serum (10%), L-Glutamine (1%), and penicillin–streptomycin (1%); the MCF-7 cell line was maintained in phenol red-free DMEM (Invitrogen) with fetal bovine serum (10%), L-Glutamine (1%), and penicillin–streptomycin (1%); Vero and MDA-MB-231 cell lines were maintained in phenol red-containing DMEM (Invitrogen) with fetal bovine serum (10%) and penicillin–streptomycin (1%); the A459 cell line was maintained in phenol red-containing DMEM (Invitrogen) with fetal bovine serum (10%), L-Glutamine (1%), and penicillin–streptomycin (1%); the Huh7 cell line was maintained in low-glucose, phenol red-containing DMEM (Invitrogen) with fetal bovine serum (10%) and penicillin–streptomycin (1%); and the RWPE1 cell line was maintained in keratinocyte–SFM serum-free medium (Invitrogen) with L-Glutamine, EGF, BPE, and penicillin–streptomycin. All cell cultures were incubated at 37 °C under a humidified atmosphere of 5% CO_2_ and 95% air. For all experiments, cells were grown in 6- and/or 96-well culture-treated microtiter plates (Techno Plastic Product AG, Trasadingen, Switzerland) with the appropriate ligand (AA) in triplicates for 72 h. MTS (CellTitre 96 Aqueous One Solution and CellTitre 96 Non-Radioactive Cell Proliferation Assays, Promega, Madison, WI, USA), MTT (475989-10GM, EMD Millipore Corp., Burlington, MA, USA) and PI/TX-100 (Invitrogen, by ThermoFisher Scientific, Waltham, MA, USA) were employed to determine cell viability per the manufacturers’ instructions. Briefly, cells were seeded in 96-well flat-bottom microtiter plates (transparent) at a density of 4500 cells (LNCaP, C4-2B, 22Rv1, DU-145, MDA-MB-231, MDA-MB-453, Huh7, MCF-7, HepG2, and Vero) per well (100 μL), and incubated for 24 h to allow the cells to adhere and resume exponential growth. After this, the wells were drained and then treated with eight concentrations of AA (5, 50, 250, 500, 1000, 2500, 5000, and 10,000 μM) and incubated for 72 h. After the incubation time, 20 μL and 10 μL of MTS and MTT reagents, respectively, were added to each well of the microtiter plates containing treated cells, untreated cells (negative control) and cell culture medium—the blank (background control) and incubated for 2 h 30 min (MTS) and 3 h (MTT). At the expiration of 2 h 30 min, absorbance was immediately read off using the iTecan plate reader at 490 nm. For MTT assay, growth media–MTT reagent suspension was aspirated after 3 h incubation period, and replaced with 100 μL DMSO, and then incubated for 15 min to completely dissolve formazan (insoluble crystal) resulting from the reductive action of NADPH-dependent dehydrogenase on tetrazolium salt of MTT. After this, the absorbance was measured using the iTecan plate reader at 570 nm. The effects of treatment on cell proliferation and survival were expressed as:

$$\mathrm{Percentage}\;\mathrm{cell}\;\mathrm{viability}\;=\;\frac{OD\;of\;treatmnet-OD\;of\;background\;control}{OD\;of\;negative\;control-OD\;of\;background\;control}\times 100$$

where OD = optical density.

For PI/TX-100 viability assay [34,35] which works by inducing cell membrane damage in viable cells, and uses PI and TX-100 to assess viability [36,37,38,39], cells were seeded in 96-well flat-bottom microtiter plates (black) at a density of 4500 cells (22Rv1, C4-2B, DU-145, LNCaP, HepG2, Huh-7, MCF-7, MDA-MB-231, MDA-MB-453, and Vero) per well (100 μL), and incubated for 24 h to allow cells to adhere and resume exponential growth. After this, growth media were aspirated from the wells, and cells were treated with eight concentrations of AA (10,000, 5000, 2500, 1000, 500, 250, 50, and 5 μM) and incubated for 72 h. After this, cells were washed with 100 μL PBS to remove dead cells and debris, treated with 100 μL of a solution containing PI (0.1 mg/mL in PBS) and TX-100 (0.1% *v*/*v*), incubated for 1–2 h. Fluorescence was measured using the iTecan plate reader (excitation wavelength = 530 nm, emission wavelength = 620 nm) to quantify the amount of attached viable cells. The effects of treatment on cell proliferation and survival were expressed as:

$$\mathrm{Percentage}\;\mathrm{Cell}\;\mathrm{viability}\;=\;\frac{Fi\;of\;treatment-Fi\;of\;background\;control}{Fi\;of\;negative\;control-Fi\;of\;background\;control}\times 100$$

where Fi = fluorescence intensity.

IC_50_ values were calculated by non-linear regression (log [concentration of inhibitor] versus response (% cell viability) using the GraphPad Prism analysis software version 10 for MacBook. For the calculation of Mean IC_50_ values, triplicate runs (n = 3) were selected.

### 2.2. Cell Imaging

Live/dead cell imaging was evaluated using propidium iodide (PI) (Invitrogen by ThermoFisher Scientific) and fluorescein diacetate (FDA) (Sigma Aldrich, Saint Louis, MO, USA). For cell imaging, cells were seeded in 6-well flat-bottom microtiter plates at a density of 100,000 cells (A549, DU-145, HepG2, Huh-7, LNCaP, MCF-7, MDA-MB-231, MDA-MB-453, and Vero) per well and incubated for 24 h to allow cells to adhere and resume exponential growth. Subsequently, the wells were drained and treated with different concentrations of AA for 24 h. Live/dead cell staining was simultaneously initiated using 2 μL of the FDA and 3 μL of PI and incubated for 15 min with gentle shaking. Images were then captured at specified wavelengths (GFP: 469, 525 nm; Texas Red: 586, 647 nm).

### 2.3. Measurement of Reactive Oxygen Species

C4-2B, MCF-7, LNCaP, MDA-MB-231, and MDA-MB-453 (1 × 10^6^) cells were seeded for 24 h and treated with various concentrations of AA (0.5, 1.25, 2.5, 5.0, and 10.0 mM) or DMSO (1%). The H2DCF-DA (Life Technologies Corporation, Eugene, OR, USA) probe (10 µM) was added during treatment and incubated for 1 h. The cells were washed twice with 1 mL of ice-cold 1X phosphate-buffered saline (PBS), trypsinized, and the resulting cell suspension was transferred to microcentrifuge tubes. The cells were then centrifuged at 500× *g* for 5 min, after which the supernatant was carefully aspirated and discarded. For flow cytometry analysis, the tubes containing the stained cells were resuspended in 200 μL of 5% fetal bovine serum (FBS) and subsequently placed on ice. DCF fluorescence was detected by FACS, data were processed using FlowJo software version 11, and mean DCF fluorescence intensities of triplicate samples were compiled.

### 2.4. Measurement of Lipid Peroxidation

Lipid peroxidation was measured in MDA-MB-453 and SK-HEP-1 cells. Accordingly, MDA-MB-453 and SK-HEP-1 (1 × 10^6^) cells were seeded for 24 h and treated with various concentrations of AA (0.5–5 mM) or DMSO (1%). The BODIPY^TM^ 581/591 C11 (Invitrogen by ThermoFisher Scientific, Waltham, MA, USA) probe (2 µM) was added during treatment and incubated for 6 h. The cells were washed twice with 1 mL of ice-cold 1X phosphate-buffered saline (PBS), trypsinized, and the resulting cell suspension was transferred to microcentrifuge tubes. The cells were centrifuged at 500× *g* for 5 min, after which the supernatant was carefully aspirated and discarded. For flow cytometry analysis, the tubes containing the stained cells were resuspended in 200 μL of 5% fetal bovine serum (FBS) and subsequently placed on ice. Fluorescence was detected by FACS, data were processed using FlowJo software, and mean fluorescence intensities of triplicate samples were compiled.

### 2.5. Western Blot Analysis

Western blot analysis of the PDHK1 protein was conducted utilizing HepG2 cells. The cells (200,000 cells per well) were seeded and allowed to incubate for 24 h, followed by treatment with various concentrations of AA (75, 150, 300, and 600 µM), and subsequently incubated for an additional 72 h. Following incubation, cell lysis was performed using RIPA buffer (120 µL) (VWR, VWRVN653-100ML) containing a protease inhibitor (Fisher Thermo, A32955, Hanoi, Vietnam). The resultant cell lysates were scraped, collected, and vortexed for 15 sec, followed by sonication for 60 sec. The lysate was then centrifuged at 14,000× *g* rpm for 10 min, and the supernatants were collected. The protein concentrations of the lysates were determined using the absorption method with a bicinchoninic acid (BCA) assay kit (BioVision Inc, Milpitas, CA, USA, K813-2500) in an iTecan plate reader. Based on the results obtained from the BSA assay, the lysates were normalized to achieve equal protein concentrations, and 40 µg of each lysate was loaded into the wells of a TGX MIDI 4–20% gel (Biorad, Hercules, CA, USA, cat. 5671093) and run at 150 V for 70 min. The gel was subsequently transferred onto a Turbo PDVF membrane (Biorad, 1704273), and after blocking with 5% BSA for 1–2 h, the membrane was incubated overnight with primary antibodies PDHK1 (1:1000, Cat #.3820, Cell Signaling Technology, Danvers, MA, USA) and anti-GAPDH (Aldrich-Sigma, Saint Louis, MO, USA). On the following day, the membrane underwent washing with TBST for three intervals of 5 min; a secondary antibody (LiCOR) was added, and the membrane was incubated with agitation for one hour. After washing with TBST for three intervals of 5 min, bands were quantified utilizing the Odyssey CLx Imaging System (iS Image Studio Ver 5.2).

### 2.6. Cell Cycle Analysis

Human prostate cancer cell lines: 22Rv1, DU-145, and LNCaP (1 × 10^6^ cells) were seeded for 24 h and treated with various concentrations of AA (0.25–1 mM) or DMSO (1%) for 24 h. The cells underwent two washes with 1 mL of ice-cold 1X phosphate-buffered saline (PBS), trypsinized, and the resultant cell suspension was transferred to microcentrifuge tubes. The cells were centrifuged at 500× *g* for 5 min, after which the supernatant was carefully aspirated and discarded. Subsequently, nucleic acid labeling commenced by fixing the cells with 1 mL of ice-cold 70% ethanol, added dropwise to the cell pellet while vortexing; the samples were then stored on ice for a minimum of 30 min. Following this fixation, ethanol was meticulously removed by centrifugation at 500× *g* for 5 min without disturbing the cell pellet, and the cells were subsequently washed in 1 mL of 1X PBS. A second removal of PBS was conducted via centrifugation at the same parameters. The cells were then resuspended in 200 μL of the staining solution, which was freshly prepared by mixing 10 mL of 1X PBS with 200 μL of propidium iodide and 200 μL of RNase A for every 20 samples being processed; this staining solution was shielded from light exposure. For flow cytometry analysis, the tubes containing the stained cells were incubated in the dark for 30 min at 37 °C and placed on ice. The analysis of the cell cycle was conducted using a CytoFLEX S Flow Cytometer (Beckman Coulter, Brea, CA, USA), with data processed utilizing FlowJo software (version 11).

### 2.7. Measurement of Apoptosis

The early stage of apoptosis was measured using Alexa Fluor^TM^ 488 Annexin V/Dead Cell Apoptosis Kit (Invitrogen, by ThermoFisher Scientific). Briefly, HepG2, LNCaP, and MDA-MB-231 cells (5 × 10^5^) were seeded for 24 h and treated with various concentrations of AA (250, 500, 1000, 2000 and 3000 µM), or DMSO (1%) for 72 h. Cells were harvested and stained with 100 µL of 1X Annexin buffer, Alexa Fluor 488 Annexin V (5 µL) and PI (1 µL) for 15 min in the dark. After incubation, 100 µL of 1X Annexin buffer was added to each sample and analyzed using a CytoFLEX S Flow Cytometer (Beckman Coulter), with data processed utilizing FlowJo software (version 11).

### 2.8. Statistical Analysis

The analysis of the data generated from this study was performed using GraphPad Prism version 10 for MacBook (www.graphpad.com; GraphPad, San Diego, CA, USA). The mean IC_50_ difference between the groups was compared using the student *t*-test and one-way analysis of variance (ANOVA). Bars represent the mean ± SD of triplicates (n = 3). Groups that differ significantly at *p* < 0.05, *p* < 0.01, *p* < 0.001 *p* < 0.0001 are indicated by *, **, *** and ****, respectively; pairwise comparison lines represent compared groups.

## 3. Results

### 3.1. Anti-Proliferative Effects of AA in Cell Cultures

It has been established that redox-active agents, such as MTS and MTT, that measure cellular metabolic activity, are not suitable for the determination of the effect of reducing agents, such as AA, on cell viability [40]. The replacement of the cell culture media with a fresh drug-free media prior to the MTT/MTS assay has been adopted previously to circumvent this incompatibility [15,16]. In this study, we referred to these methods as the “pre-exposure (PE) method” and the “post-drainage (PD) method”. While the PE method involves removing the cell culture medium containing AA 24 h after treatment, followed by replacing it with fresh culture medium devoid of AA and incubating for 48 h before initiating the MTS/MTT assay, the PD method involves removing the cell culture medium containing AA 72 h after treatment, followed by replacing it with fresh culture medium devoid of AA and incubating for 1–2 h before initiating the MTS/MTT assay. These methods are different from the standard method of incubation, which we referred to as the “constant exposure (CE) method”. This method involves incubating the cells with AA for 72 h before initiating the MTS/MTT assay. We compared these methods with an alternative fluorometric assay (PI/TX-100). We found that these methods gave inconsistent results compared to results obtained with PI/TX-100 assay (Figures S1 and S2A,B). Specifically, the PE method yielded IC_50_ values of 996.61 ± 4.20 μM (MTS) and 1153.00 ± 5.03 μM (MTT) in A549 compared to PI/TX-100, which yielded a better IC_50_ value of 459.96 ± 2.10 μM (Figures S1 and S2A). In addition, the CE method yielded an IC_50_ value of >10,000 μM (MTS) and 1238.00 ± 3.45 μM (MTT), which are higher than that of PD method of 980.24 ± 3.12 μM (MTS) and 1077.00 ± 2.65 μM (MTT). The estimated IC_50_ values for the PD method in MTS and MTT assays were significantly higher than those of PI/TX-100 (451.40 ± 1.39 μM) (Figures S1 and S2B).

**Figure 1:**
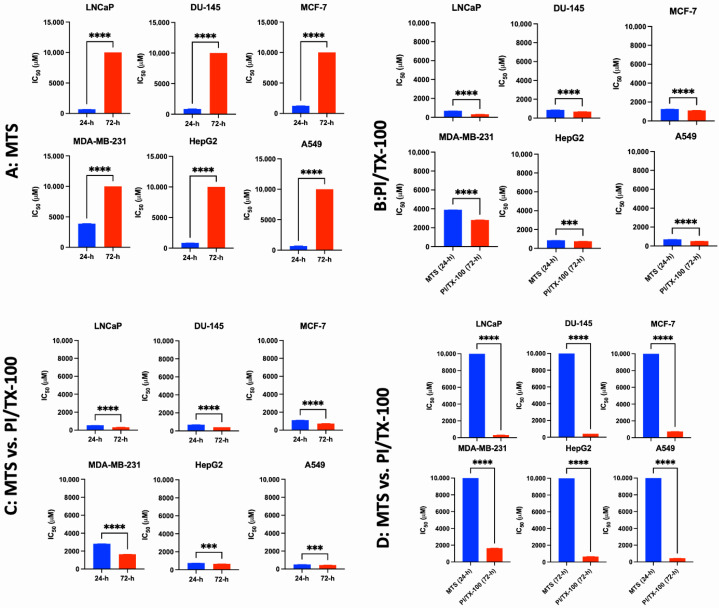
Statistical comparison of the pre and constant exposure conditions in validating the anticancer activity of AA in cancer cell lines. Pre-exposure condition: Cell cultures were treated with AA for 24 h, replaced with fresh media and incubated for 48 h before adding MTS/PI-TX-100 reagent. Constant Exposure: Cell cultures were treated with AA and incubated for 72 h before adding MTS/PI-TX-100 reagent. (**A**): Comparison of 24 h pre-exposure and 72 h constant exposure using MTS assay. (**B**): Comparison of 24 h pre-exposure and 72 h constant exposure using PI/TX-100 assay. (**C**): Comparison of 24 h pre-exposure condition using MTS and PI/TX-100 assays. (**D**): Comparison of 72 h constant exposure conditions using MTS and PI/Tx-100 assays. ***: *p* < 0.001, ****: *p* < 0.0001.

We compared the IC_50_ values obtained for various cell lines using MTS adopting the PE and CE methods to further validate the preferred incubation methods (Figure 1A–D, Table S1). Our results revealed that the IC_50_ values for the PE method were significantly lower than those of the CE method (>1000 μM) as evaluated by MTS assay (Figure 1A). This observation further validates the involvement of AA in forming formazan, an indication of cell proliferation. We further compared the IC_50_ values obtained for the PE and CE methods using PI/TX-100 assay. Our data revealed that the IC_50_ values for the PE method were higher than those of the CE method (Figure 1B). The differences in IC_50_ values might be due to the exposure time (24 and 72 h incubation of cell cultures with AA). To further validate the preference of PI/TX-100 over MTS, we compared the IC_50_ values between MTS and PI/TX-100 assays for the PE method (Figure 1C) and CE method (Figure 1D). Our results showed that the PI/TX-100 assay consistently quantified the IC_50_ values of AA in cell cultures compared to the MTS assay. Moreover, removing AA from cell culture media 24 h before the initiation of the MTS assay did not completely resolve the problem; thus, making it an unreliable strategy to overcome the issues associated with determining the cytotoxicity index of AA in cell cultures using MTS assay. This evidence further reinforces our decision to use the PI/TX-100 assay to assess the anticancer effect of AA in cell cultures. The observed inconsistency in the MTS/MTT assays might be due to the ability of AA to convert the tetrazolium salt of MTS/MTT to formazan, an indication of cell proliferation (Figure S3). Thus, it obscures the actual anticancer activity of AA in cell cultures. Taken together, the PI/TX-100 assay is well-suited for screening the anticancer activity of AA and other bioactive compounds that can interfere with formazan formation.

Based on these data, we employed the PI/TX-100 fluorometric assay. Using this method, we determined the antiproliferative activity of AA on different cell lines: LNCaP, DU-145, C4-2B, 22Rv1, MCF-7, MDA-MB-231, MDA-MB-453, HepG2, Huh7, SK-HEP-1, A549, Vero, RWPE1 (Table 1 and Table S1). The data showed that AA inhibited the growth of the tested cancer cells (LNCaP, DU-145, C4-2B, 22Rv1, MCF-7, MDA-MB-231, MDA-MB-453, HepG2, Huh7, SK-HEP-1, and A549) compared to normal cells (Vero and RWPE1). Among the represented prostate cancer cell lines, 22Rv1 and C4-2B cells were more predisposed to AA-induced toxicity compared to DU-145 and LNCaP cells. The degree of antiproliferative effect of AA, based on the experimental IC_50_ values, was in the order of DU-145 < LNCaP < 22Rv1 ≈ C4-2B. Among breast cancer cells, MDA-MB-453 was the most responsive, followed by MCF-7 and MDA-MB-231 cells. SK-HEP-1 was most responsive among liver cancers, followed by HepG2 and Huh7 cells. The results further divulged that AA inhibited the growth of NSCLC (A549) cells. Compared to cancer cells, normal cells (Vero and RWPE1) were not severely impacted by high concentrations of AA. Vero (normal kidney epithelial cells) was more resistant than RWPE1 (normal prostate epithelial cells).

Among the tested cells, prostate and breast cancer cells are more sensitive to AA treatment. Except for DU-145, these cells depend on nuclear receptors, androgen receptor (AR) and estrogen receptor alpha (ERα) for their viability [41,42,43,44]. MDA-MB-231, which does not express ERα and expresses very little AR, is the least sensitive to AA among breast cancer cells [45]. A459, the only lung cancer cell that we tested, has been shown to express AR [46]. Moreover, the responsiveness of the liver cancer cells that we tested also closely reflects their AR expression status [47,48]. Vero and RWPE1 cells were least impacted by high concentrations of AA compared to cancer cells. Possible explanations for the limited effects of AA in Vero and RWPE1 cells may include the possession of robust enzymic and non-enzymic antioxidant defense mechanisms, such as higher levels of catalase and glutathione peroxidase, which efficiently neutralize H_2_O_2_ in these cells compared to cancer cells, thereby mitigating oxidative damage [49]; differential expression of AA transporters (sodium-dependent vitamin C transporters [SVCTs] and GLUTs) [50]; and enhanced DNA repair mechanisms in normal cells [27,51].

**Figure 2:**
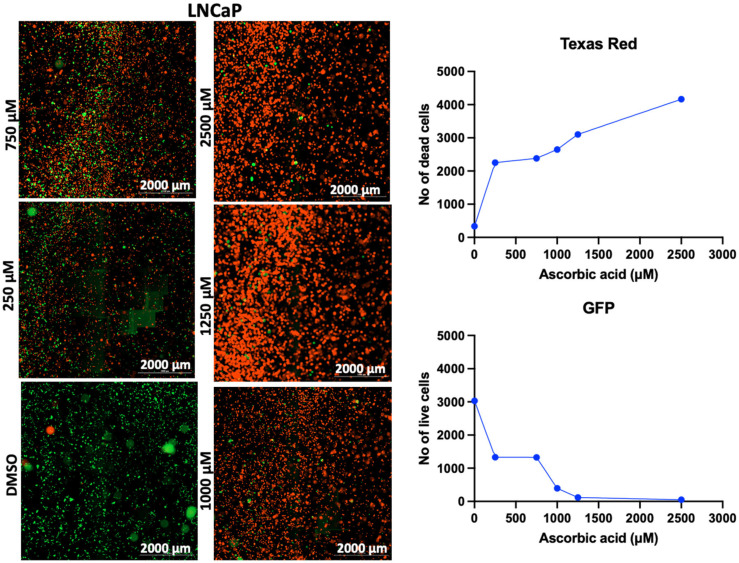
Effects of different concentrations of AA on the proliferation of LNCaP cells. Cells were seeded for 24 h, treated with various concentrations of AA and further incubated for 24 h. Cells were stained with FDA/PI, and imaged at the threshold (GFP: 469 and 525 nm; Texas Red: 586 and 647 nm).

**Figure 3:**
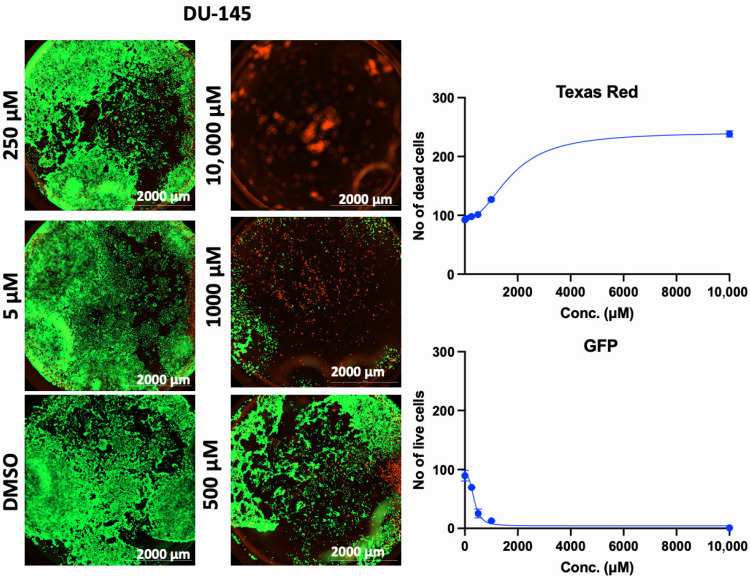
Effects of different concentrations of AA on the proliferation of DU-145 cells. Cells were seeded for 24 h, treated with various concentrations of AA and further incubated for 24 h. Cells were stained with FDA/PI, and imaged at the threshold (GFP: 469 and 525 nm; Texas Red: 586 and 647 nm).

**Figure 4:**
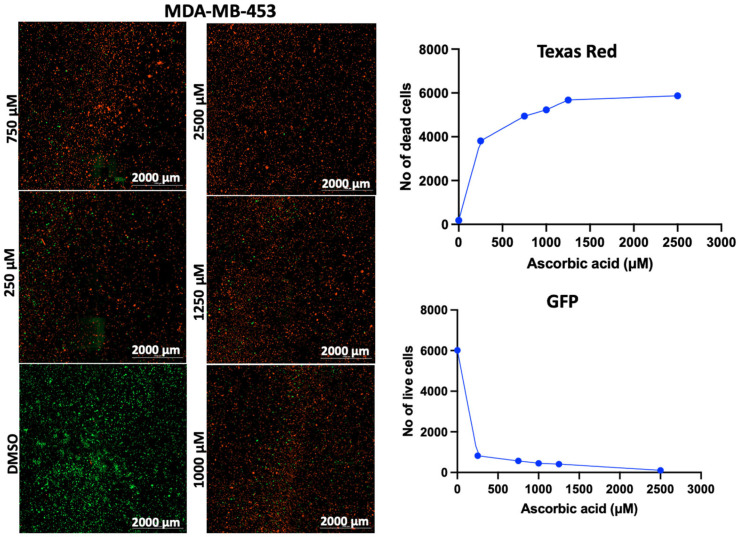
Effects of different concentrations of AA on the proliferation of MDA-MB-453 cells. Cells were seeded for 24 h, treated with various concentrations of AA and further incubated for 24 h. Cells were stained with FDA/PI, and imaged at the threshold (GFP: 469 and 525 nm; Texas Red: 586 and 647 nm).

**Figure 5:**
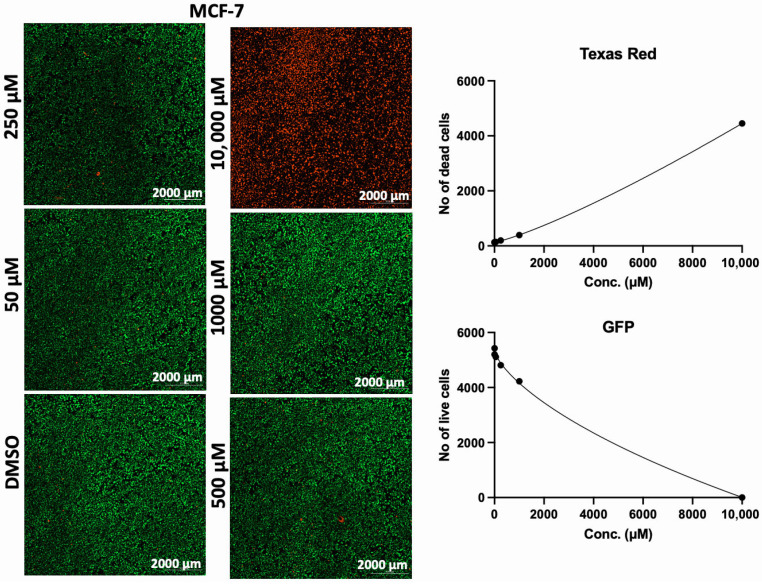
Effects of different concentrations of AA on the proliferation of MCF-7 cells. Cells were seeded for 24 h, treated with various concentrations of AA and further incubated for 24 h. Cells were stained with FDA/PI, and imaged at the threshold (GFP: 469 and 525 nm; Texas Red: 586 and 647 nm).

**Figure 6:**
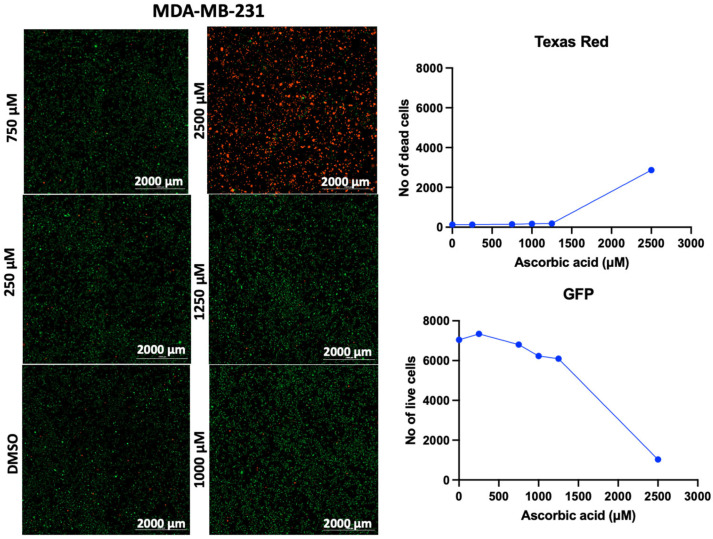
Effects of different concentrations of AA on the proliferation of MDA-MB-231 cells. Cells were seeded for 24 h, treated with various concentrations of AA and incubated for 24 h. Cells were stained with FDA/PI, and imaged at the threshold (GFP: 469 and 525 nm; Texas Red: 586 and 647 nm).

**Figure 7:**
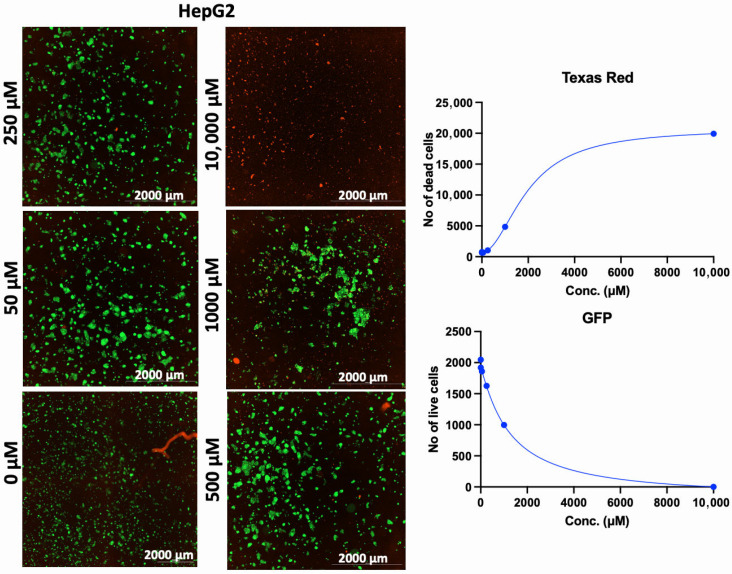
Effects of different concentrations of AA on the proliferation of HepG2 cells. Cells were seeded for 24 h, treated with various concentrations of AA and further incubated for 24 h. Cells were stained with FDA/PI, and imaged at the threshold (GFP: 469 and 525 nm; Texas Red: 586 and 647 nm).

**Figure 8:**
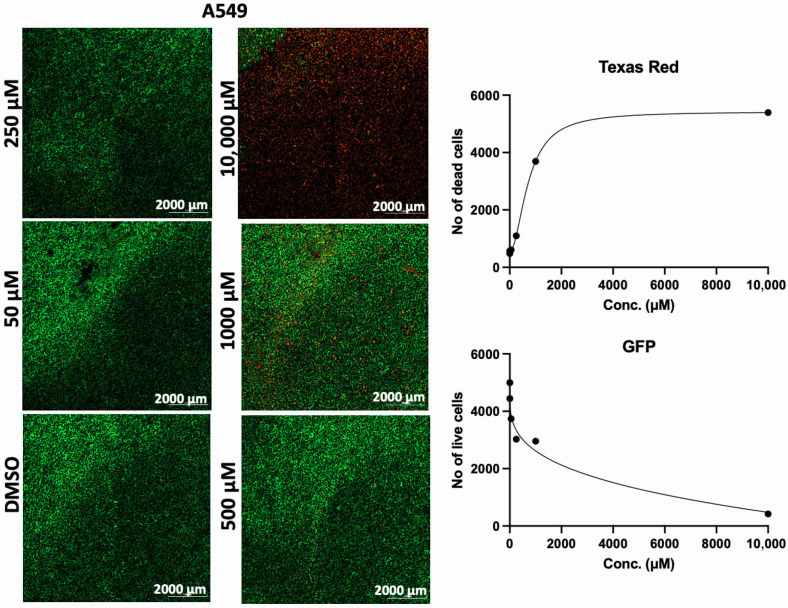
Effects of different concentrations AA on the proliferation of A549 cells. Cells were seeded for 24 h, treated with various concentrations of AA and further incubated for 24 h. Cells were stained with FDA/PI, and imaged at the threshold (GFP: 469 and 525 nm; Texas Red: 586 and 647 nm).

**Figure 9:**
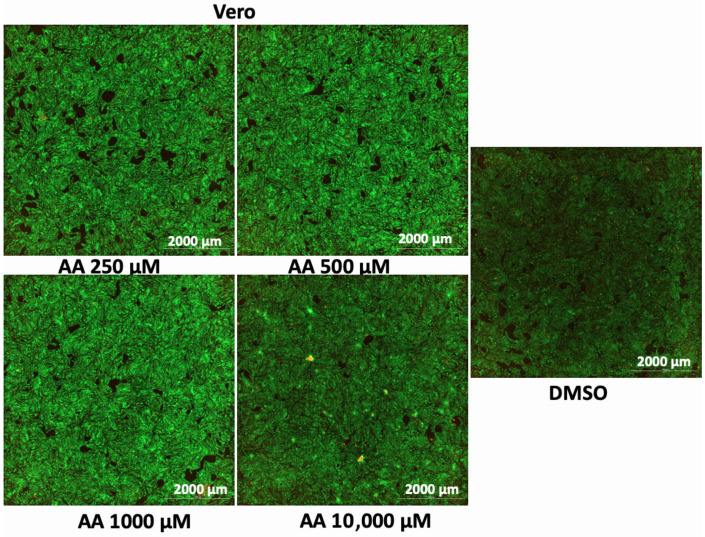
Effects of different concentrations AA on the proliferation of Vero cells. Cells were seeded for 24 h, treated with various concentrations of AA and further incubated for 24 h. Cells were stained with FDA/PI, and imaged at the threshold (GFP: 469 and 525 nm; Texas Red: 586 and 647 nm).

In addition to the PI/TX-100 assay, we explored the live/dead cell imaging using FDA/PI and the extracellular ATP measurement using the RealTime-Glo™ Extracellular ATP assay reagent, to validate the antiproliferative effect of AA in cell cultures. For the live/dead cell imaging experiments, we selected LNCaP, MDA-MB-453, HepG2, MCF-7, A549, DU-145, and Vero cell lines. These cell lines were treated with various concentrations of AA for 24 h and incubated with FDA and PI. The results revealed that AA inhibited the growth of cancer cells (HepG2, MCF-7, A549, and DU-145) while being relatively less cytotoxic to Vero cells (Figure 2, Figure 3, Figure 4, Figure 5, Figure 6, Figure 7, Figure 8 and Figure 9). The region stained green depicts live cells (green fluorescent protein, GFP), while the region stained red depicts dead cells (Texas Red).

Additionally, our results showed an increase in the accumulation of extracellular ATP levels following exposure of MDA-MB-453 cells to AA (Figure S4). This finding suggests that AA preferentially targets cancer cells, leading them to apoptosis while preserving the growth of normal cells. The results validated the observed anticancer activity of AA in cell cultures using the PI/TX-100.

### 3.2. AA Induces Cytotoxicity Through the Generation of ROS and Lipid Peroxidation in Cancer Cells

**Figure 10:**
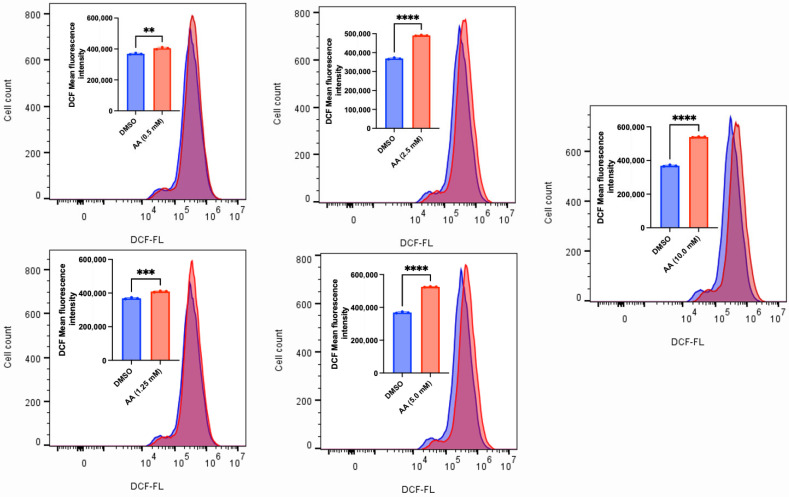
AA mediates ROS generation in MDA-MB-453 breast cancer cells. MDA-MB-453 (1 × 10^6^) cells were seeded for 24 h and treated with various concentrations of AA (0.5, 1.25, 2.5, 2.5, 5.0, and 10.0 mM), or DMSO (1%). H2DCF-DA probe (10 µM) was added during treatment and incubated for 1 h. DCF fluorescence was detected by FACS, and mean DCF fluorescence intensities of triplicate samples were compiled. ** *p* < 0.01, *** *p* < 0.001, ****: *p* < 0.0001.

**Figure 11:**
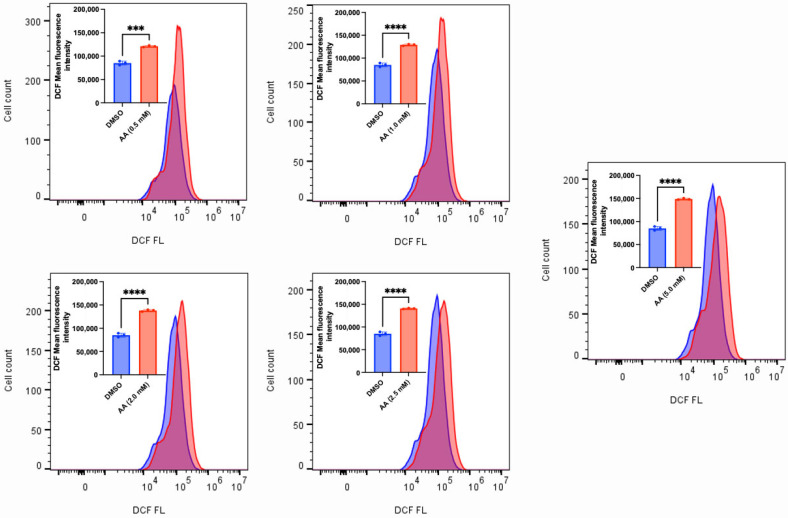
AA mediates ROS generation in C4-2B prostate cancer cells. C4-2B (1 × 10^6^) cells were seeded for 24 h and treated with various concentrations of AA (0.5, 1.0, 2.0, 2.5, and 5.0 mM), or DMSO (1%). H2DCF-DA probe (10 µM) was added during treatment and incubated for 1 h. DCF fluorescence was detected by FACS, and mean DCF fluorescence intensities of triplicate samples were compiled. *** *p* < 0.001, ****: *p* < 0.0001.

**Figure 12:**
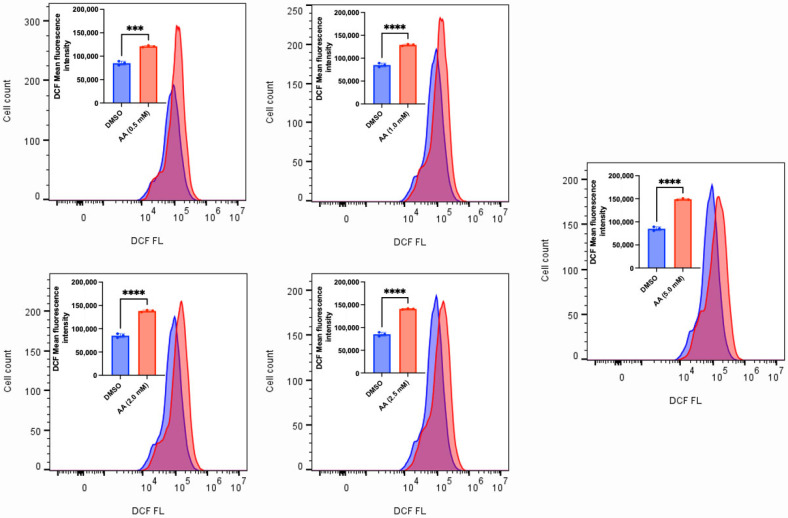
AA mediates ROS generation in LNCaP prostate cancer cells. LNCaP (1 × 10^6^) cells were seeded for 24 h and treated with various concentrations of AA (0.5, 1.0, 2.0, 2.5, and 5.0 mM), or DMSO (1%). H2DCF-DA probe (10 µM) was added during treatment and incubated for 1 h. DCF fluorescence was detected by FACS, and mean DCF fluorescence intensities of triplicate samples were compiled. *** *p* < 0.001, ****: *p* < 0.0001.

**Figure 13:**
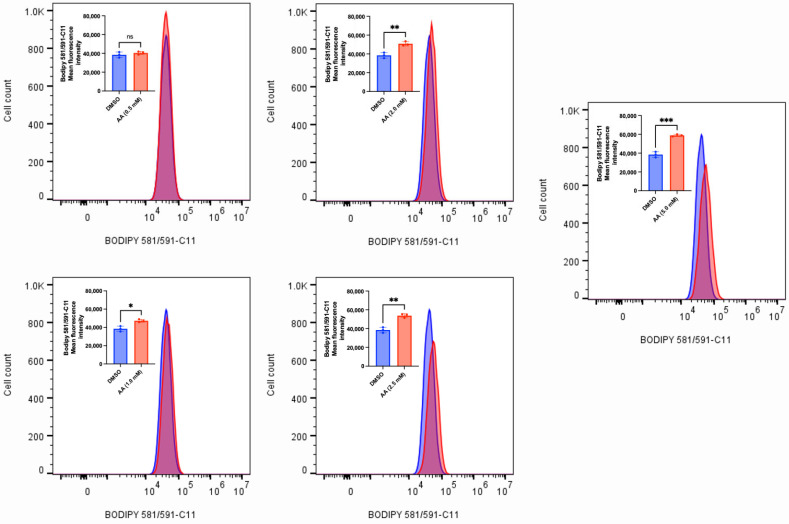
AA induces lipid peroxidation in MDA-MB-453 breast cancer cells. MDA-MB-453 (1 × 10^6^) cells were seeded for 24 h and treated with various concentrations of AA (0.5, 1.0, 2.0, 2.5, and 5.0), or DMSO (1%). BODIPY 581/591-C11 probe (2 µM) was added during treatment and incubated for 6 h. Fluorescence was detected by FACS, and mean fluorescence intensities of triplicate samples were compiled. * *p* < 0.05, ** *p* < 0.01, *** *p* < 0.001, ns: not significant.

**Figure 14:**
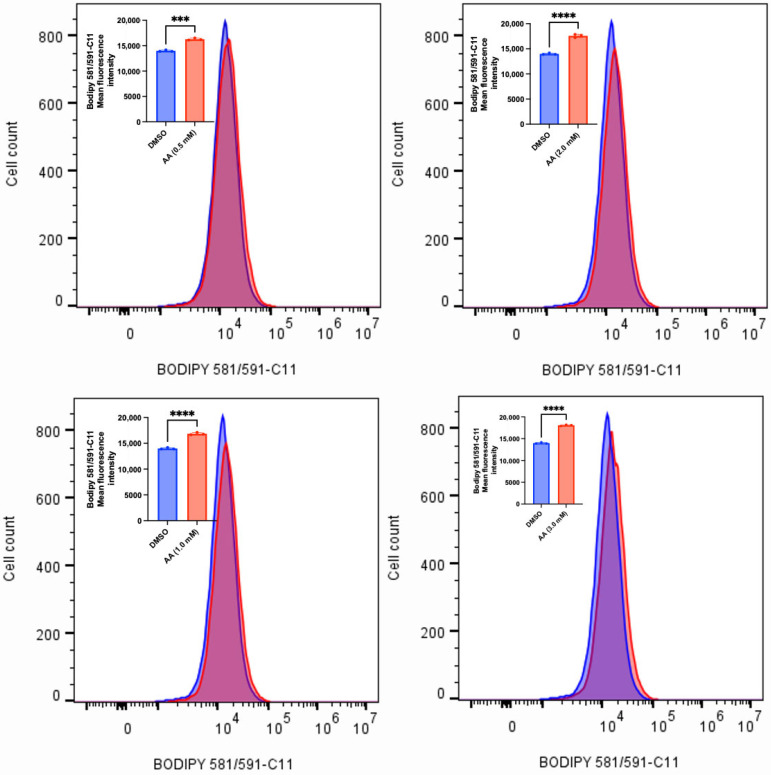
AA induces lipid peroxidation in SK-HEP-1 liver cancer cells. SK-HEP-1 (1 × 10^6^) cells were seeded for 24 h and treated with various concentrations of AA (0.5, 1.0, 2.0, 3.0 mM), or DMSO (1%). BODIPY 581/591-C11 probe (2 µM) was added during treatment and incubated for 6 h. Fluorescence was detected by FACS, and mean fluorescence intensities of triplicate samples were compiled. *** *p* < 0.001, ****: *p* < 0.0001.

AA has long been known to confer antioxidant protection in normal cells. While this is true at lower concentrations, recent findings have revealed that administering high concentrations of AA can induce the generation of ROS [15,52,53]. The sustained accumulation of ROS could overwhelm the antioxidant defense mechanisms, thus leading to cell death. To validate this observation, we exposed MDA-MB-453, C4-2B, LNCaP and MCF-7 (some of the responsive cancer cells) and MDA-MB-231 (the less responsive cancer cell) to various concentrations of AA. We observed that AA induced a concentration-dependent generation of ROS in MDA-MB-253, C4-2B and LNCaP cells (Figure 10, Figure 11 and Figure 12). Incubation of MDA-MB-231 and MCF-7 cells with AA at 5.0 mM also induced the generation of ROS (Figure S5). The accumulation of ROS in cells can overwhelm the antioxidant defense mechanism, thereby triggering lipid peroxidation and altering the integrity of membrane lipids [54]. To validate this, we measured the level of lipid peroxidation in AA-treated MDA-MB-453 and SK-HEP-1 cells using a C11-BODIPY probe. Our results showed that AA significantly induced the generation of lipid peroxides at the tested concentrations (Figure 13 and Figure 14). Taken together, AA mediates the generation of ROS, which, when accumulated, can induce lipid peroxidation—an indication of ferroptosis.

### 3.3. Catalase and Exogenous Iron Counteract the Anticancer Activity of AA

**Figure 15:**
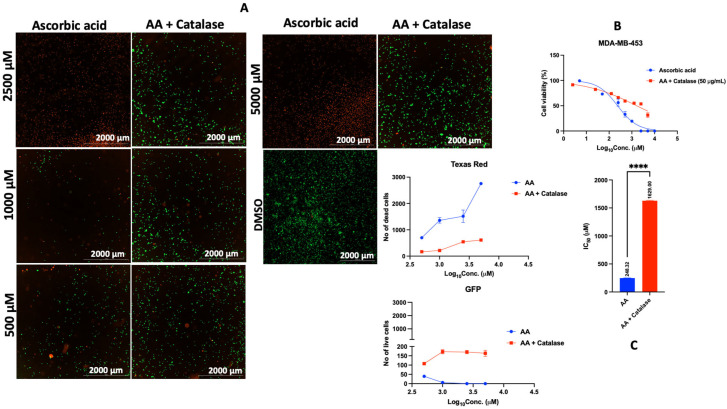
Effect of catalase on the antiproliferative activity of AA in MDA-MB-453 cells. (**A**): MDA-MB-453 cells were seeded for 24 h, treated with various concentrations of AA and incubated for 24 h. Cells were stained with FDA/PI and imaged at the threshold (GFP: 469 and 525 nm, Texas Red: 586 and 647 nm). (**B**,**C**): MDA-MB-453 cells were seeded for 24 h. After 24 h, cells were treated with fresh medium containing various concentrations of AA (5–10,000 μM). For combination therapy, the cells were treated with various concentrations of AA at a fixed concentration of catalase (50 μg/mL), and incubated for 72 h. The viability of MDA-MB-453 cells was assessed by PI/TX-100. IC_50_ was calculated by non-linear regression (log [Conc. of drug] versus response (% (T-B/C-B)) using the GraphPad Prism analysis software. ****: *p* < 0.0001.

**Figure 16:**
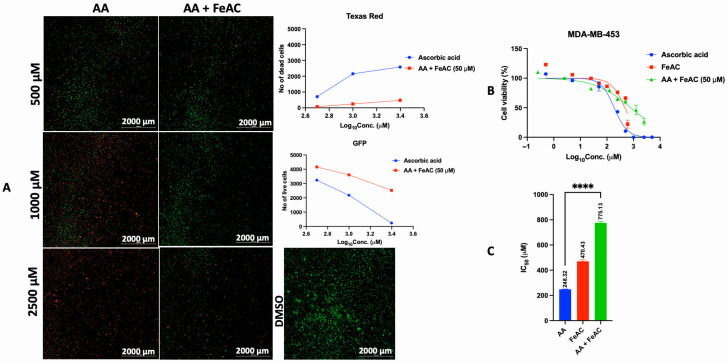
Effect of exogenous iron on the antiproliferative activity of AA in MDA-MB-453 cells. (**A**): Cells were seeded for 24 h, treated with various concentrations of AA and incubated for 24 h. Cells were stained with FDA/PI and imaged at the threshold (GFP: 469 and 525 nm, Texas Red: 586 and 647 nm). (**B**,**C**): Cells were seeded for 24 h. After 24 h, cells were treated with fresh medium containing various concentrations of AA (5–10,000 μM), and FeAC (0.5–600 μM). For combination therapy, the cells were co-treated with various concentrations of AA at a fixed concentration of FeAC (50 μM), and incubated for 72 h. The viability of MDA-MB-453 cells was assessed by PI/TX-100. IC_50_ was calculated by non-linear regression (log [Conc. of drug] versus response (% (T-B/C-B)) using the GraphPad Prism analysis software. ****: *p* < 0.0001.

**Figure 17:**
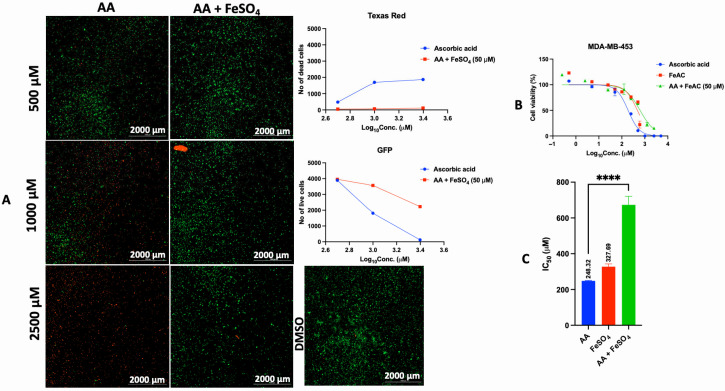
Effect of exogenous iron on the antiproliferative activity of AA in MDA-MB-453 cells. (**A**): Cells were seeded for 24 h, treated with various concentrations of AA and incubated for 24 h. Cells were stained with FDA/PI and imaged at the threshold (GFP: 469 and 525 nm, Texas Red: 586 and 647 nm). (**B**): Cells were seeded for 24 h. After 24 h, cells were treated with fresh medium containing various concentrations of AA (5–10,000 μM), and FeSO_4_ (0.5–600 μM). For combination therapy, the cells were treated with various concentrations of AA at a fixed concentration of FeSO_4_ (50 μM), and incubated for 72 h. The viability of MDA-MB-453 cells was assessed by PI/TX-100. (**C**): IC_50_ was calculated by non-linear regression (log [Conc. of drug] versus response (% (T-B/C-B)) using the GraphPad Prism analysis software. ****: *p* < 0.0001.

Catalase is an antioxidant enzyme that converts H_2_O_2_ to water [55,56]. The antiproliferative effect of AA is through the generation of ROS in the form of H_2_O_2_ [15]. Based on this evidence, we co-incubated cancer cells (MDA-MB-453) with AA and catalase. Our results show that catalase counteracted the antiproliferative effect of AA, possibly by scavenging AA-mediated ROS production in MDA-MB-453 cells (Figure 15A–C). This effect could be due to the ability of catalase to degrade H_2_O_2_ generated during the redox recycling of AA. The role of iron is crucial for AA-mediated redox recycling. For instance, the conversion of AA to ascorbate radical is facilitated by the reduction of iron III (Fe^3+^) to iron II (Fe^2+^), which, in the process, molecular oxygen (O_2_) is reduced to superoxide anion radical (O_2_^.^), and then H_2_O_2_. The generation of H_2_O_2_ and subsequent accumulation in the tumor cells is maintained at a physiological concentration of iron (as contained in cell culture media). However, exogenous iron sources (ferrous sulfate and ferric sulfate) decompose H_2_O_2_, generating short-lived hydroxyl radicals that cannot permeate the cell membrane of MDA-MB-453 cells in culture. Consequently, H_2_O_2_ cannot diffuse into the intracellular space where it can conveniently induce the production of hydroxyl radicals, which can damage proteins, lipids, and nucleic acids inside the cells, leading to cell death. This process reduces the instant toxicity of AA to tumor cells [29,57,58]. Going by this literature precedence, we co-exposed cells to various concentrations of AA at fixed concentrations of iron (FeAC and FeSO_4_), and our results showed that iron subverted the antiproliferative effect of AA in MDA-MB-453 (Figure 16A–C and Figure 17A–C) and LNCaP cells (Figures S6 and S7), likely due to its decomposition of H_2_O_2_.

### 3.4. AA Inhibits Colony Formation in Cancer Cells

**Figure 18:**
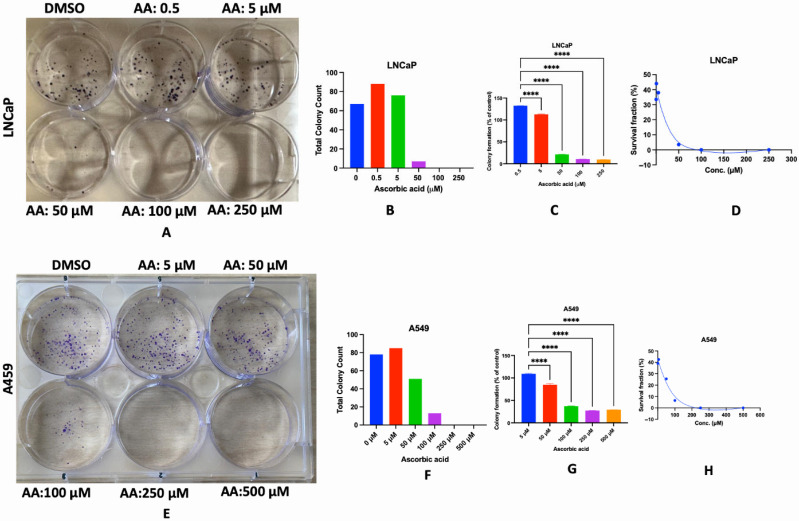
Effect of AA on colony formation ability of LNCaP and A549 cells. LNCaP and A549 cells (200 cells/well) were seeded for 24 h. After 24 h, cells were treated with fresh medium containing various concentrations of AA (5–500 μM) for 72 h, followed by replacement with a fresh medium devoid of AA, and incubated for another 72 h. Cell culture media were replaced after 72 h until distinct colonies were established (between 6–10 days). After day 7, the cells were washed with PBS, fixed with paraformaldehyde (PFA, 4%) for 20 min and stained with crystal violet dye for 20 min. The dye was then washed with distilled water, dried for 24 h and images were taken thereafter (**A**,**E**). The total number of colonies was estimated using a stereomicroscope (**B**,**F**) or spectrophotometrically at 560 nm (**C**,**G**), and the percentage survival fraction was calculated relative to the control (**D**,**H**). ****: *p* < 0.0001.

The alteration of colony formation represents a mechanism by which anticancer agents inhibit tumor growth, as it signifies the capacity of cancer cells to proliferate indefinitely and establish macroscopic colonies [59]. Numerous chemotherapeutic agents, along with radiation and targeted therapies, impede this clonogenic potential; thus, indicating long-term cytotoxic or cytostatic effects on tumor cells [60,61]. We investigated the effect of AA on the colony formation ability of LNCaP and A549 cells. These cells were selected because of their ability to adhere to the culture plates after careful washings. We observed that the exposure of these tumor cells to AA prevented colony formation. The results revealed that 50 to 250 μM of AA prevented colony formation in LNCaP (Figure 18A–D), while 100 to 500 μM prevented colony formation in A549 cells (Figure 18E–H).

### 3.5. AA Downregulates PDHK1 to Drive ROS Generation

**Figure 19:**
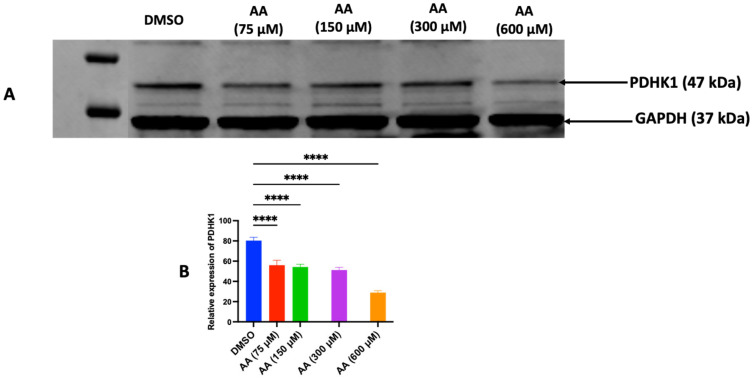
Downregulation of PDHK1 is associated with tumor cell death. (**A**) Western blot analysis of PDK1 protein expression from AA-treated HepG2 cell line. (**B**) Mean values of normalized protein expression of PDK1. ****: *p* < 0.0001.

**Figure 20:**
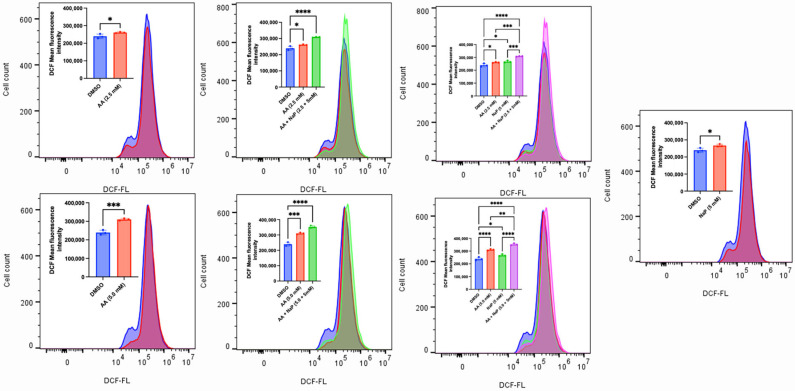
The Combination of AA and pyruvate enhances ROS generation in MDA-MB-453 cells. MDA-MB-453 (1 × 10^6^) cells were seeded for 24 h and treated with various concentrations of AA (2.5 and 5.0 mM), sodium pyruvate: NaP (5 mM), AA + NaP (2.5 + 5 mM), AA + NaP (5.0 + 5 mM), or DMSO (1%). H2DCF-DA probe (10 µM) was added during treatment and incubated for 1 h. DCF fluorescence was detected by FACS, and mean DCF fluorescence intensities of triplicate samples were compiled in part. * *p* < 0.05, ** *p* < 0.01, *** *p* < 0.001, **** *p* < 0.0001.

Upregulation of pyruvate dehydrogenase kinase 1 (PDHK1) is vital in the growth and progression of several tumors [62,63]. We found that the exposure of hepatic tumor cells (HepG2) to AA resulted in a concentration-dependent downregulation of PDHK1 (Figure 19A,B). These findings suggest that the anticancer activity of AA could be derived in part from the stimulation of respiratory metabolism due to activation of the activity of the pyruvate dehydrogenase complex. Furthermore, by suppressing the expression of this protein, the activity of pyruvate dehydrogenase complex (PDHC) is enhanced; thus, triggering the continual conversion of pyruvate to acetyl-CoA, which is fed into the Kreb cycle to generate energy currency (ATP/GTP) and reducing equivalents—which are needed to switch on the electron transport chain (ETC). The augmented activity of the ETC may result in heightened production of ROS, predominantly in the form of O_2_^−^•, as a consequence of electron leakage at complexes I and III [64,65,66]. In pursuit of this objective, we subjected MDA-MB-453 cells to AA and pyruvate treatment. Our findings revealed that the combination of pyruvate and AA elevated the overall levels of ROS in MDA-MB-453 cells (Figure 20).

### 3.6. Effect of AA on Cell Cycle Progression

**Figure 21:**
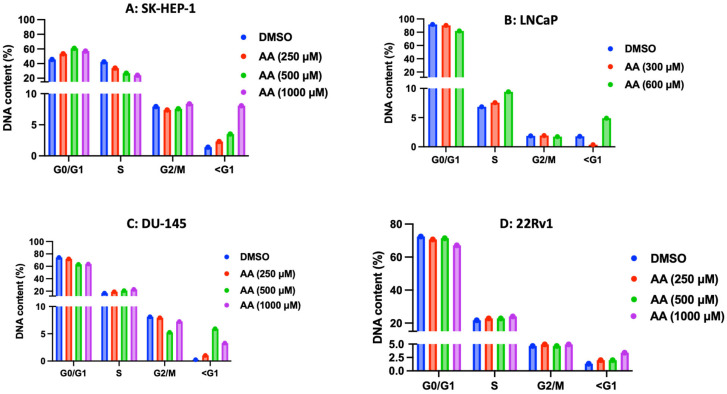
Cell cycle analysis of (**A**) SK-HEP-1, (**D**) 22Rv1, (**B**) LNCaP, and (**C**) DU-145 cells. Representative histogram data of the cell cycle analysis of SK-HEP-1, LNCaP, DU-145, and 22Rv1 cells treated with various concentrations of AA or DMSO (1%) for 24 h. <G1 cells represent apoptotic cells.

The anticancer activity of AA is also evidenced by its perturbation of cell cycle progression in representative cancer cells [67,68,69]. To investigate if AA induces a similar effect on the cell cycle of different cancer cell lines, we probed its impact on cell cycle progression in SK-HEP-1, LNCaP, 22Rv1 and DU-145 cells. We treated each cell line with graded concentrations of AA for 24 h. For the vehicle (DMSO), the data shows that most SK-HEP-1 cells are in the G1 phase (46.6%), 42.3% in the S phase, and 7.93% in the G2 phase, indicating a high level of cells actively synthesizing DNA. The percentage of cells in the G1 phase increases as the concentration of AA increases. It rises from 46.6% in the DMSO control to 53.4% at 250 µM, and 60.8% and 57.1% at 500 and 1000 µM, respectively. This increase suggests that AA induces G1 phase cell cycle arrest in SK-HEP-1 cells. G1 arrest is a common effect of many anticancer agents, as it often reflects the activation of checkpoints that halt the cell cycle in response to cellular stress or DNA damage. The percentage of cells in the S phase (DNA synthesis phase) decreases as the concentration of AA increases: from 42.3% in the DMSO control to 33.9% at 250 µM, 26.3% at 500 µM, and further to 24.1% at 1000 µM. This reduction in the S-phase cell population indicates that AA may inhibit cell cycle progression into the S phase, possibly due to a blockade at the G1/S transition point. The percentage of cells in the G2 phase remains relatively stable across all conditions, with a slight increase at 1000 µM (8.06%) compared to the control (7.93%). This suggests that AA does not significantly affect the G2/M checkpoint but may have a minor role in influencing the G2 phase, particularly at higher concentrations. The sub-G1 population, which typically represents apoptotic cells, increases significantly as the concentration of AA increases: from 1.39% in the DMSO control to 2.31% at 250 µM, 3.52% at 500 µM, and 8.06% at 1000 µM. The rising sub-G1 population indicates that higher AA concentrations may induce apoptosis in SK-HEP-1 cells. This pro-apoptotic effect may be related to oxidative stress or cellular damage caused by high concentrations of AA (Figure 21A and Figure S8A–D. We further compared the impact of AA on the cell cycle progression of several prostate cancer cell lines, including LNCaP, 22Rv1, and DU-145 cells. Unlike in SK-HEP-1 cells, where AA induces G1-phase arrest, we observed AA-mediated S-phase arrest in LNCaP, 22Rv1 and DU-145. For the vehicle (DMSO), the data showed that most LNCaP cells were in the G1 phase (91.8%), 6.86% in the S phase, and 1.87% in the G2 phase. However, treatment with 300 µM AA slightly reduced the DNA content in the G1 phase (90.3%) and sub-G1 population (0.35%), while increasing the DNA content in the S phase (7.57%) and G2 phase (1.93%). When the concentration of AA was doubled (that is 600 µM), the DNA content in the G1-phase further dropped (82.0%), while increasing that of the S phase (9.42%) with a marked rise in sub-G1 (4.90%), indicating enhanced S-phase arrest at this higher concentration (Figure 21B and Figure S9A–C).

Furthermore, AA had a similar effect on DU-145 cells. For the vehicle (DMSO), the data showed that most DU-145 cells are in the G1 phase (74.3%), 16.8% in the S phase, and 8.12% in the G2 phase. Treatment of DU-145 cells with 250 µM AA slightly decreased DNA content in the G1 phase (72.0%) and increased the DNA content in the S phase (19.0%) compared to the control. The DNA content in the G2 phase remains relatively stable (7.93%), while the sub-G1 fraction rises slightly (1.03%), suggesting minor apoptotic cell death. Increasing the concentration of AA to 500 μM decreased the proportion of DNA content in the G1 population (63.2%) and G2 phase (6.26%) but increased the DNA content in the S phase (20.8%). The sub-G1 content increases substantially to 5.92%, indicating an increase in apoptotic cell death at this concentration. An additional increase in the concentration of AA (1000 µM) did not significantly affect the proportion of cells in the G1 phase (63.7%), S phase (22.9%), and G2 phase (7.24%) compared to 500 µM (Figure 21C and Figure S10A–D). This implies that AA induces a cell cycle arrest in the S-phase in DU-145 cells, especially at higher concentrations (500 µM and 1000 µM). The effects suggest that AA may promote S-phase accumulation and cell death in a dose-dependent manner, with a peak effect observed at the 500 µM concentration.

In addition, the effect of AA on cell cycle progression was also tested in 22Rv1 cells. For the vehicle (DMSO), the data showed that most 22Rv1 cells are in the G1 phase (72.5%), 21.7% in the S phase, and 4.65% in the G2 phase. However, 22Rv1 cells treated with AA at 250 µM exhibited a slight decrease in the G1 phase (70.7%) and a slight increase in the S phase (22.8%), G2 phase (4.94%) and a concomitant increase in the sub-G1 population (1.99%). This suggests a minor shift in cells from the G1 to the S phase, indicating a mild effect on cell cycle progression. The increase in AA concentration to 500 µM slightly altered the proportions of cells in the G1 phase (71.4%) and S phase (22.8%) but did not affect the proportion of cells in the G2 phase (4.65%). However, as the concentration of AA was doubled (1000 µM), the proportion of cells in the G1 phase (67.1%) is reduced with a concomitant increase in the proportions of cells in the S phase (24.0%), G2 phase (4.94%), along with a rise in the sub-G1 population (3.39%) compared to the control (Figure 21D and Figure S11A–D. This indicates that AA induces S-phase cell cycle arrest in 22RV1 cells in a similar manner to its effects on LNCaP and DU-145 cells. In contrast, AA causes a G1 phase cell cycle arrest in SK-HEP-1 cells.

### 3.7. AA Induces Apoptosis in Cancer Cells

**Figure 22:**
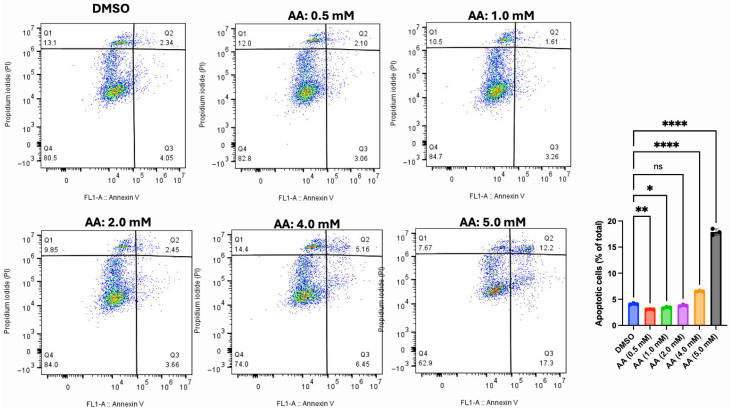
Effect of AA on apoptosis induction in LNCaP cells. LNCaP (5 × 10^5^) cells were seeded for 24 h and treated with various concentrations of AA (0.5, 1.0, 2.0, 4.0, and 5.0 mM), or DMSO (1%) for 48 h. Cells were harvested and stained with Alexa Fluor 488 Annexin V (5 µL) and PI (1 µL) for 15 min, then 100 µL of 1X Annexin buffer was added and analyzed using a CytoFLEX S flow cytometer. * *p* < 0.05, ** *p* < 0.01, **** *p* < 0.0001, ns: Not significant.

**Figure 23:**
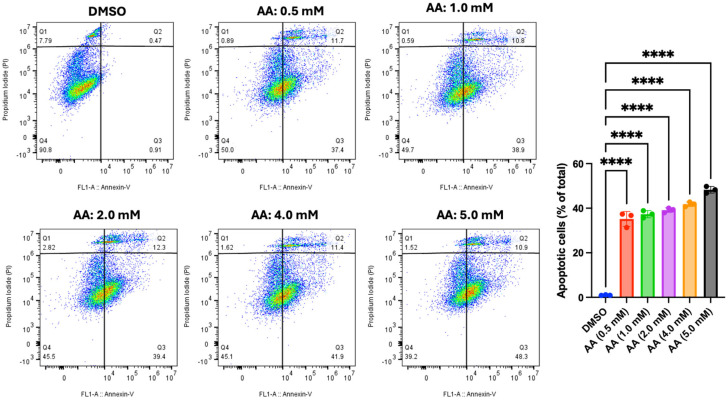
Effect of AA on apoptosis induction in MDA-MB-453. MDA-MB-453 (5 × 10^5^) cells were seeded for 24 h and treated with various concentrations of AA (0.5, 1.0, 2.0, 4.0, and 5.0 mM), or DMSO (1%) for 48 h. Cells were harvested and stained with Alexa Fluor 488 Annexin V (5 µL) and PI (1 µL) for 15 min, then 100 µL of 1X Annexin buffer was added and analyzed using a CytoFLEX S flow cytometer. **** *p* < 0.0001.

**Figure 24:**
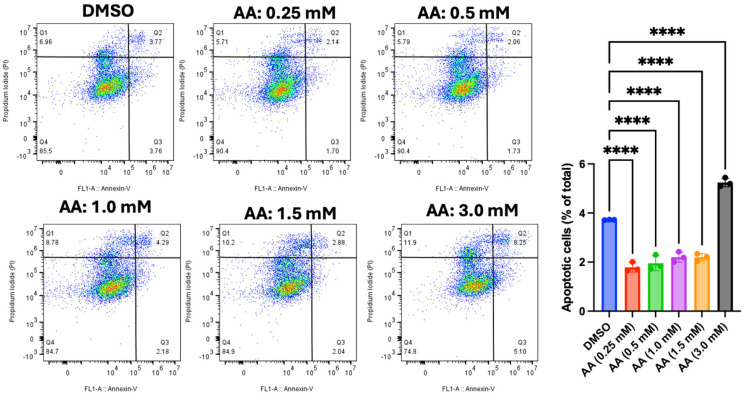
Effect of AA on apoptosis induction in HepG2. HepG2 (5 × 10^5^) cells were seeded for 24 h and treated with various concentrations of AA (0.25, 0.5, 1.0, 2.0, and 3.0 mM), or DMSO (1%) for 48 h. Cells were harvested and stained with Alexa Fluor 488 Annexin V (5 µL) and PI (1 µL) for 15 min, then 100 µL of 1X Annexin buffer was added and analyzed using a CytoFLEX S flow cytometer. **** *p* < 0.0001.

The flow cytometry data described above revealed evidence of apoptosis induction by AA in all tested cell lines. Hence, we used Annexin V/PI to further confirm the mechanism of cell death in MDA-MB-453 (breast cancer cell), LNCaP (prostate cancer cell), and HepG2 (liver cancer cell) cells. For the vehicle (DMSO, 1%), the data showed that most LNCaP cells (64.3%) are in the Q4 (viable cell region), while 5.77% are undergoing the early stage of apoptosis (Q3). However, treatment with AA significantly increased the proportions of cells undergoing early stages of apoptosis, specifically 6.70% (at 0.5 mM of AA), 9.03% (at 1.0 mM of AA), 13.1% (at 2.0 mM of AA), 13.0% (at 4.0 mM of AA), and 21.7% (at 5.0 mM of AA) (Figure 22). This observation further validates the outcome from cell cycle analysis, where higher doses of AA increased the proportion of apoptotic cells in <G1 phase of the cell cycle. In addition, we confirmed our observation using MDA-MB-453 (one of the cells that was mostly susceptible to AA toxicity). For the vehicle (DMSO, 1%), the data showed that most MDA-MB-453 cells (90.80%) are in Q4 (viable cell region), while 0.91% are undergoing the early stage of apoptosis (Q3). However, treatment with AA significantly increased the proportions of cells undergoing early stages of apoptosis, specifically 37.40 % (at 0.5 mM of AA), 38.90% (at 1.0 mM of AA), 39.40% (at 2.0 mM of AA), 41.90% (at 4.0 mM of AA), and 48.30% (at 5.0 mM of AA) (Figure 23). Further confirmation was carried out using HepG2 cells. Our data showed that AA induced the early stages of apoptosis at a concentration of 3 mM in HepG2 cells (Figure 24). Below this concentration, AA did not orchestrate the early stages of apoptosis.

## 4. Discussion

AA is a vital micronutrient with antioxidant, pro-oxidant, immunomodulatory, and epigenetic properties that have been linked to tumor regression for over seven decades. Plasma AA levels are consistently lower in cancer patients compared to healthy individuals [18,70,71]. In murine model, genetic inhibition of L-gulono-γ-lactone oxidase, a rate-limiting enzyme in the biosynthesis of AA, resulted in hematopoietic dysfunction [72,73,74]. These findings reinforce AA’s involvement in tumor biology. Early studies by Benade, Cameron, Campbell, and Pauling [19,20,21,22,23,24] revealed AA’s selective tumor-inhibitory effects as a monotherapy. Notably, normal cells tolerate higher AA concentrations, potentially due to more stable NF-κB (RelB) expression. In contrast, AA-induced ROS in cancer cells downregulates RelB, reducing SIRT3 and MnSOD levels, thereby amplifying oxidative and metabolic stress in cancer cells [75]. This vulnerability presents a therapeutic opportunity for selective cancer targeting.

In our study, we evaluated AA’s impact on diverse tumor cell lines and highlighted the limitations of commonly used redox-sensitive assays, specifically MTS and MTT, in assessing AA’s anticancer activity. These tetrazolium-based colorimetric assays yielded inconsistent results, particularly when AA was present during treatment. AA’s reductive capacity can non-enzymatically convert MTS/MTT tetrazolium salts to formazan, falsely indicating cell proliferation. This interference, first reported by Chakrabarti et al. [40], compromises assay reliability. Although some researchers attempt to remove AA prior to MTS/MTT application [15,16], residual AA may still skew results. Conversely, a confirmatory assay using FDA/PI (Figure S12) demonstrated the limitations of these redox-sensitive assays.

Against this backdrop, we employed the PI/TX-100 assay, a colorimetric method combining propidium iodide (PI) and Triton X-100 (TX-100), to quantify the effect of AA on the viability of diverse cancer and normal cell lines. PI is a fluorescent dye that is permeable only to the membrane of dead or dying cells, while TX-100 permeabilizes cell membranes [37]. The fluorescence shift in PI upon DNA binding enables accurate assessment of cell viability. This method circumvents the redox interference seen with MTS/MTT and is suitable for evaluating reducing agents like AA. Alternative assays such as Alamar Blue and SRB have also been used, measuring metabolic activity and protein content, respectively [76,77]. Using PI/TX-100, we assessed AA’s effects on ten cancer cell lines (LNCaP, DU-145, C4-2B, 22Rv1, MDA-MB-231, MDA-MB-453, MCF-7, HepG2, A549, Huh7) and two normal lines (Vero, RWPE1).

We observed that AA elicits differing effects on the viability of the tested cells, with Huh7 being the least susceptible among the cancer cells we tested. Based on experimental IC_50_ values, the degree of the susceptibility of the other tested cancer cells is in the order DU-145 < LNCaP < 22Rv1 ≈ C4-2B (prostate cancers); MDA-MB-231 < MCF-7 < MDA-MB-453 (breast cancers); Huh7 < HepG2 < SK-HEP-1 (liver cancers), Vero < RWPE1 (normal cells). The result further revealed that AA inhibited the growth of A549 cells, the only lung cancer cell line we tested. The resistance of Huh7 could stem from its robust antioxidant defenses, particularly peroxiredoxin II (Prx II) [78], and its glycolytic metabolic profile [79], which may mitigate AA-induced oxidative stress. These features collectively explain the resistance of Huh7 cells, and it may be common to other AA-resistant cancer cells. Fluorescence imaging, ATP quantification, and clonogenic assays confirmed AA-induced cell death in cancer cells. Notably, many responsive lines expressed high levels of AR, consistent with prior findings [80]. Although we observed a correlation between AA sensitivity and nuclear hormone receptor expression, direct validation of AR/ER levels was not performed. Future studies should include receptor profiling and functional assays to clarify their role in AA responsiveness.

**Figure 25:**
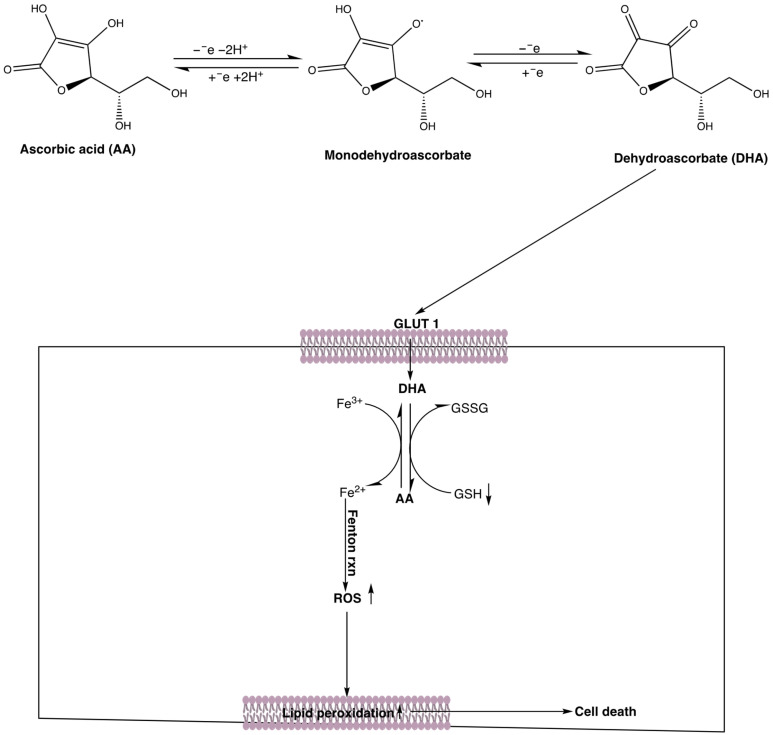
AA metabolism and toxicity mechanisms in tumor cells. The oxidation of AA to mono- and de-hydroascorbate leads to the generation of H_2_O_2_, which, upon accumulation, exerts cytotoxic effects in tumor cells. Dehydroascorbate binds to GLUT 1 and is transported into the cells, where it is subsequently reduced back to ascorbate at the expense of GSH. This reduction in GSH levels concomitantly increased ROS production, initiating processes such as DNA damage and lipid peroxidation, ultimately culminating in apoptosis. Created with ChemDraw (Revvity Signals Inc., Waltham, MA, USA, Version 22) and BioRender (Toronto, ON, Canada) by Uche Arunsi.

AA’s cytotoxicity is largely mediated by ROS generation at high concentrations ^10-14^. In the presence of labile iron, AA is oxidized to dehydroascorbate (DHA), releasing H_2_O_2_. DHA enters cells via glucose transporters and is reduced back to AA, depleting glutathione (GSH) and increasing ROS burden [15]. Excess ROS damages lipids, proteins, and DNA, leading to cell death (Figure 25). AA’s tumor cell-selectivity may depend on SVCT/GLUT transporter expression and antioxidant capacity, supported by literature [50,75] and our findings in Vero and RWPE1 cells. Future work should include transporter profiling, uptake studies, and antioxidant enzyme comparisons to elucidate these mechanisms.

Catalase and exogenous iron modulate AA’s anticancer effects by scavenging or altering ROS dynamics [56]. In MDA-MB-453 cells, catalase reversed AA-induced cytotoxicity, likely by degrading H_2_O_2_. Similarly, iron co-treatment reduced AA’s efficacy, possibly by generating short-lived hydroxyl radicals that fail to penetrate cell membranes [29,57,58]. While these results offer phenotypic insights, molecular targets remain unidentified. Proteomic and transcriptomic analyses, along with assays for mitochondrial function and DNA damage, could clarify downstream effects of ROS modulation. Moreover, future research should employ ROS-specific probes (e.g., Amplex Red for H_2_O_2_, MitoSOX for superoxide, HPF for hydroxyl radicals) and enzymatic assays to map oxidative stress across cell lines. This would help determine the contribution of other ROS types to AA-induced cancer cell-selective cytotoxicity.

Pyruvate dehydrogenase kinase 1 (PDHK1) serves an essential function in tumor growth and progression by modulating cellular metabolism, particularly the Warburg effect, a metabolic adjustment observed in cancer cells wherein glucose metabolism is redirected towards glycolysis, even in the presence of oxygen (aerobic glycolysis) [81,82,83]. This metabolic reconfiguration facilitates cancer cells’ survival, proliferation, and evasion of apoptosis. PDHK1 executes the phosphorylation and subsequent inactivation of pyruvate dehydrogenase (PDH), thereby inhibiting the conversion of pyruvate to acetyl-CoA and obstructing mitochondrial oxidative phosphorylation (OXPHOS). As a result, cancer cells must depend on an anaerobic source of energy, glycolysis, even under oxygen-rich environments, to promote swift ATP production and the biosynthesis of macromolecules imperative for tumor growth. PDHK1 expression is upregulated by hypoxia-inducible factor 1-alpha (HIF-1α) [33,82], a prominent regulator of the hypoxic response in tumors. Under hypoxic conditions, which are frequently encountered in solid tumors, PDHK1 aids in redirecting metabolism toward glycolysis (Warburg effect); thus, diminishing oxygen consumption and enhancing tumor viability. The glycolytic shift enhances the availability of metabolic intermediates necessary for the synthesis of nucleotides, amino acids, and lipids crucial for rapid tumor proliferation. The inhibition of PDH by PDHK1 decreases the production of ROS from the mitochondria [84,85], thereby preventing apoptosis induced by oxidative stress.

**Figure 26:**
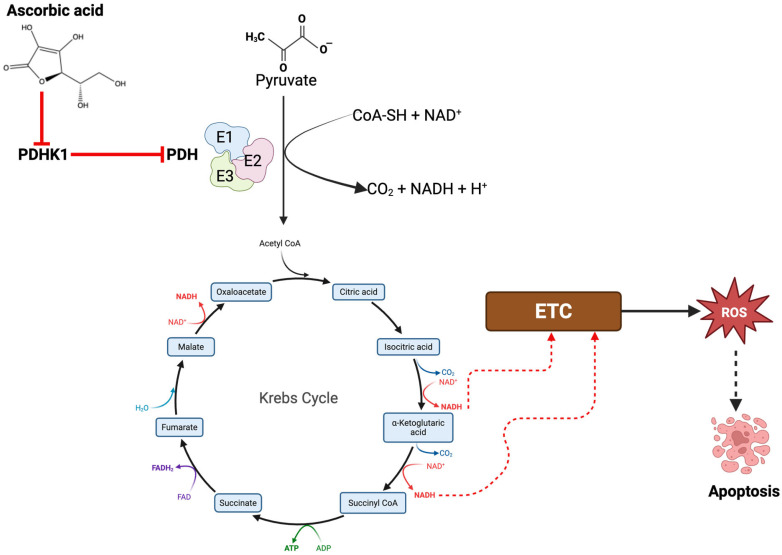
AA downregulates the expression of PDHK1. In tumor cells, PDHK1 drives glycolysis by inhibiting the activity of the pyruvate dehydrogenase complex (PDC), which is known to catalyze the conversion of pyruvate to acetyl-CoA, a precursor to the Kreb cycle and OXPHOS. In the presence of AA, the expression of PDHK1 is altered, thus facilitating the conversion of pyruvate to acetyl-CoA and ultimately driving the generation of ROS and the induction of apoptosis.

Numerous cancers leverage this pathway to circumvent chemotherapy-induced cell death. Therefore, PDHK1 has emerged as a promising therapeutic target, considering its vital contribution to cancer metabolism [86]. In the present study, we established that AA downregulated the protein levels of PDHK1. AA may induce PDHK1 downregulation due to its repressive effect on HIF-1α levels [87,88,89]. The consequence of PDHK1 downregulation is the reactivation of PDH, which compels cancer cells to revert to mitochondrial respiration, leading to increased ROS production and subsequent apoptosis (Figure 26). Co-treatment with AA and pyruvate enhanced ROS generation in MDA-MB-45 cells, suggesting that AA reverses the Warburg effect by reactivating mitochondrial OXPHOS, thereby sensitizing cancer cells to oxidative stress. This metabolic reprogramming strategy represents a promising therapeutic avenue for targeting tumor cell survival.

Although our data show a concentration-dependent downregulation of PDHK1 following AA treatment in HepG2 cells and a corresponding increase in ROS levels in MDA-MB-453 cells co-treated with AA and pyruvate, we recognize that these findings are correlative. The mechanistic link between PDHK1 suppression and ROS production remains to be fully clarified. PDHK1 is known to inhibit mitochondrial oxidative phosphorylation by inactivating the pyruvate dehydrogenase complex, thereby decreasing mitochondrial ROS output. Therefore, PDHK1 downregulation may restore mitochondrial respiration and lead to ROS accumulation [84,85]. While others have shown that repression of PDHK1 triggers ROS generation [90], future studies using PDHK1 knockdown or overexpression models, combined with ROS measurement and cell viability assays, are necessary to more concretely establish causality. These approaches would help determine whether PDHK1 directly mediates AA-induced oxidative stress and cytotoxicity in cancer cells.

When not scavenged, ROS accumulation in cells can trigger cell cycle arrest or apoptosis [91,92]. In this study, we found that high concentrations of AA modulated cell cycle events at either G1 or S phase in a cell-dependent manner, while also increasing the levels of apoptotic cells. Using LNCaP, MDA-MB-453 and HepG2 as representative cancer cells, we used Annexin V staining to confirm apoptosis induction by AA. These findings further reveal the anticancer effect of AA in vitro and align with the body of evidence suggesting that high concentrations of AA exert pro-apoptotic effects on cancer cells while preserving normal cells [27,93]. This selective cytotoxicity at high concentrations substantiates the proposition that AA may be a potential therapeutic agent in oncology, particularly in conjunction with other chemotherapeutic agents [23,94].

Our study revealed that some of the tested cancer cell lines responded to AA treatment at high concentrations. Such levels are higher than the typical AA plasma concentrations, which saturate around 220 µM after oral dosing. However, pharmacological concentrations in the millimolar range (about 13,400 µM) can be reached with intravenous administration, as shown in previous clinical studies [27,93,95]. Future research exploring combination therapies of lower AA doses with standard-of-care chemotherapeutic agents or metabolic modulators could provide insights about strategy to enhance the efficacy of AA within its plasma concentrations. Nevertheless, the higher AA IC_50_ values, relative to the conventional drugs, is consistent with its redox-based mechanisms requiring elevated extracellular concentration to generate cytotoxic levels of H_2_O_2_ in the tumor microenvironment [27,93,95]. Other redox-active agents like alpha-lipoic acid (ALA) [96] and menadione [97] show similar profiles, reinforcing AA’s therapeutic plausibility.

Our study included only hard-to-treat solid tumors (prostate, breast, liver and lung cancers) [98], but lacked hematological and AA-resistant tumors. Future research should incorporate leukemia, lymphoma, and resistant variants to broaden applicability and identify resistance mechanisms. This expansion will enhance translational relevance and help identify patient populations most likely to benefit from AA-based therapies.

## 5. Conclusions

We revealed herein that AA elicits distinct anti-proliferative effects on various human cancers (HepG2, Huh-7, LNCaP, DU-145, C4-2B, 22Rv1, MCF-7, MDA-MB-231, MDA-MB-453, and A549) and normal cell lines (Vero and RWPE1), which may be linked to the AR expression status of the cancer cell lines. Additional evidence of the anticancer effect of AA was demonstrated using live/dead cell imaging, extracellular ATP measurement, quantification of ROS, ferroptosis, apoptosis, measurement of the expression levels of PDHK1, and evaluation of colony formation. Furthermore, we showed the inconsistency of the MTS and MTT assays in assessing the anticancer effect of AA, regardless of the protocol adopted in these assays, suggesting the inadequacy of the solution proposed in the literature as a panacea for this problem. We found the PI/TX-100 assay to be an efficient and cost-effective assay suitable for evaluating the antiproliferative activity of AA and possibly other reducing drugs. The connection between AR expression and cancer cell sensitivity to AA, suggested by our study and that of Wang et al. [80], could be pursued to develop novel combination therapies for AR-dependent tumors, including certain sub-types of triple negative breast cancer, and the early stage and castration-resistant phase of prostate cancer.

## Figures and Tables

**Table 1:** Determination of the anticancer activity of AA by PI/TX-100.

Cell Lines	PI/TX-100	Type of Cancers
LNCaP	317.60 ± 8.49	Prostate cancer
DU-145	413.20 ± 1.49	Prostate cancer
C4-2B	252.50 ± 10.50	Prostate cancer
22Rv1	265.70 ± 10.67	Prostate cancer
MDA-MB-453	253.10 ± 6.43	Breast cancer
MDA-MB-231	1650.00 ± 5.53	Breast cancer
MCF-7	728.70 ± 10.47	Breast cancer
SK-HEP-1	242.10 ± 10.19	Liver Cancer
HepG2	656.20 ±14.61	Liver Cancer
Huh7	5329.00 ± 8.32	Liver Cancer
A549	445.20 ± 5.35	Lung cancer
RWPE1	3120.00 ± 11.50	Normal cell
Vero	7820.00 ± 9.50	Normal cell

## Data Availability

The dataset could be available on request.

## Supplementary Materials

The following supporting information can be downloaded at: https://www.mdpi.com/article/10.3390/cancers17172877/s1. Figure S1: Exposure dependency impact of AA on A549 cells with respect to different viability (colorimetric and fluorometric) assays. Cells were seeded for 24 h, treated with fresh medium containing various concentrations of AA (5–10,000 μM) and incubated for 24 h (pre-exposure condition: PE) or 72 h (continuous exposure condition: CE). A: For 24-h PE exposure, the cells were treated for 24 h, replaced with fresh medium devoid of AA, and subsequently incubated for 48 h. B: For 72 CE, cells were treated with AA for 72 h. 72 h CE condition was further modified into post-drainage: PD), in which case, after 72 h, the spent growth medium was aspirated and replaced with fresh medium devoid of ascorbic acid 1-2 h before the termination of the experiment. Viability was assessed by MTS or PI/TX-100. IC_50_ values were calculated by non-linear regression (log [Conc. of drug] versus response (% (T-B/C-B)) using the GraphPad Prism analysis software. **** *p* < 0.0001. Figure S2: Exposure dependency impact of AA on A549 cells with respect to different viability (colorimetric and fluorometric) assays. Cells were seeded for 24 h, treated with fresh medium containing various concentrations of AA (5 – 10000 μM) and incubated for 24 h (pre-exposure condition: PE) or 72 h (continuous exposure condition: CE). A: For 24-h PE exposure, the cells were treated for 24 h, replaced with fresh medium devoid of AA, and incubated for 48 h. B: For 72 CE, cells were treated with AA for 72 h. 72 h CE condition was further modified into post-drainage: PD), in which case, after 72 h, the spent growth medium was aspirated and replaced with fresh medium devoid of ascorbic acid 1-2 h before the termination of the experiment. Viability was assessed by MTT or PI/TX-100. IC_50_ values were calculated by non-linear regression (log [Conc. of drug] versus response (% (T-B/C-B)) using the GraphPad Prism analysis software. **** *p* < 0.0001. Figure S3: AA enhances formazan formation in the presence of MTS/MTT reagent in cell cultures. In viable cells, NADH reacts with tetrazolium salts of MTS/MTT to form formazan (purple coloration), which indicates the cell’s metabolic activity. However, in dead cells, tetrazolium salt of MTS/MTS is not reduced to formazan (yellow coloration is retained). However, in the presence of ascorbic acid (AA), the formation of formazan (purple coloration) is observed in both live/dead cells; thus, leading to a false positive result. This is because AA can mimic NADH, reducing the tetrazolium salt of MTS to formazan. *Created with Bionder.com*. Figure S4: Effect of AA on extracellular ATP levels. MDA-MB-453 (1 x 10^4^) cells were incubated for 24 h and treated with various concentrations of AA and 4X RealTime-Glo™ Extracellular ATP Assay Reagent (GA501A), and incubated for 24 h. Luminescence measurements were collected using an iTecan plate reader set at 37 °C. Figure S5: AA mediates ROS generation. MCF-7 and MDA-MB-231 (1 x 10^6^) cells were seeded for 24 h and treated with AA (5.0 mM), or DMSO (1%). H2DCF-DA probe (10 µM) was added during treatment and incubated for 1 h. DCF fluorescence was detected by FACS, and mean DCF fluorescence intensities of triplicate samples were compiled. **** *p* < 0.0001. Figure S6: Effect of exogenous iron (FeAC) on the antiproliferative activity of ascorbic acid (AA) in LNCaP cells. A: Cells were seeded for 24 h, treated with various concentrations of AA. For combination therapy, the cells were co-treated with various concentrations of AA at a fixed concentration of FeAC (50 μM) and subsequently incubated for 24 h. Cells were stained with fluorescein diacetate (FDA) and propidium iodide, and imaged at the threshold (GFP: 469 and 525 nm, Texas Read: 586 and 647 nm). Figure S7: Effect of exogenous iron (FeSO_4_) on the antiproliferative activity of ascorbic acid (AA) in LNCaP cells. A: Cells were seeded for 24 h, treated with various concentrations of AA. For combination therapy, the cells were co-treated with various concentrations of AA at a fixed concentration of FeSO_4_ (50 μM) and subsequently incubated for 24 h. Cells were stained with fluorescein diacetate (FDA) and propidium iodide, and imaged at the threshold (GFP: 469 and 525 nm, Texas Read: 586 and 647 nm). Figure S8: Cell cycle analysis of SK-HEP-1 cells. (A-D) Cell cycle analysis by flow cytometry of SK-HEP-1 cells treated with DMSO (A: 1%) or ascorbic acid (B: 250, C: 500, and D: 1000 µM). <G1 cells represent apoptotic cells. Figure S9: Cell cycle analysis of LNCaP cells. (A-C) Cell cycle analysis by flow cytometry of LNCaP cells treated with DMSO (A: 1%) or ascorbic acid (B: 300, and C: 600 µM). <G1 cells represent apoptotic cells. Figure S10: Cell cycle analysis of DU-145 cells. (A-D) Cell cycle analysis by flow cytometry of DU-145 cells treated with DMSO (A: 1%) or ascorbic acid (B: 250, C: 500, and D: 1000 µM). <G1 cells represent apoptotic cells. Figure S11: Cell cycle analysis of 22Rv1 cells. (A-D) Cell cycle analysis by flow cytometry of 22Rv1 cells treated with DMSO (A: 1%) or ascorbic acid (B: 250, C: 500, and D: 1000 µM). <G1 cells represent apoptotic cells. Figure S12: Reaction mechanisms of FDA/PI viability assays. In live cells, esterase converts FDA to fluorescein, which produces fluorescence that can be detected with GFP filter (469, 525 nm). However, in dead cells, FDA remained untransformed due to low activity of esterase. In dead cells, PI enters the nucleus, intercalates with DNA and produces fluorescence which can be detected with Texas Red filter (586 and 647 nm). Table S1. Comparison of the anticancer activity of AA at pre-exposure and constant exposure conditions Table S2. Determination of the anticancer activity of AA by MTS cell viability assay.
