# Supplementary material for: Anticancer Effects of Ascorbic Acid: Not All Sides Fit All
Source: Cancers (Basel). 2025 Sep 1;17(17):2877. doi: 10.3390/cancers17172877 (PMC12427553; doi:10.3390/cancers17172877)
Supplement: Supplementary file 1 [file cancers-17-02877-s001.zip › cancers-3799644-supplementary.pdf]

cancers

MDPI

Supplementary Materials

Anticancer Effects of Ascorbic Acid: Not All Sides Fit All

Uche O. Arunsi, Jeremiah O. Olugami and Adegboyega K. Oyelere

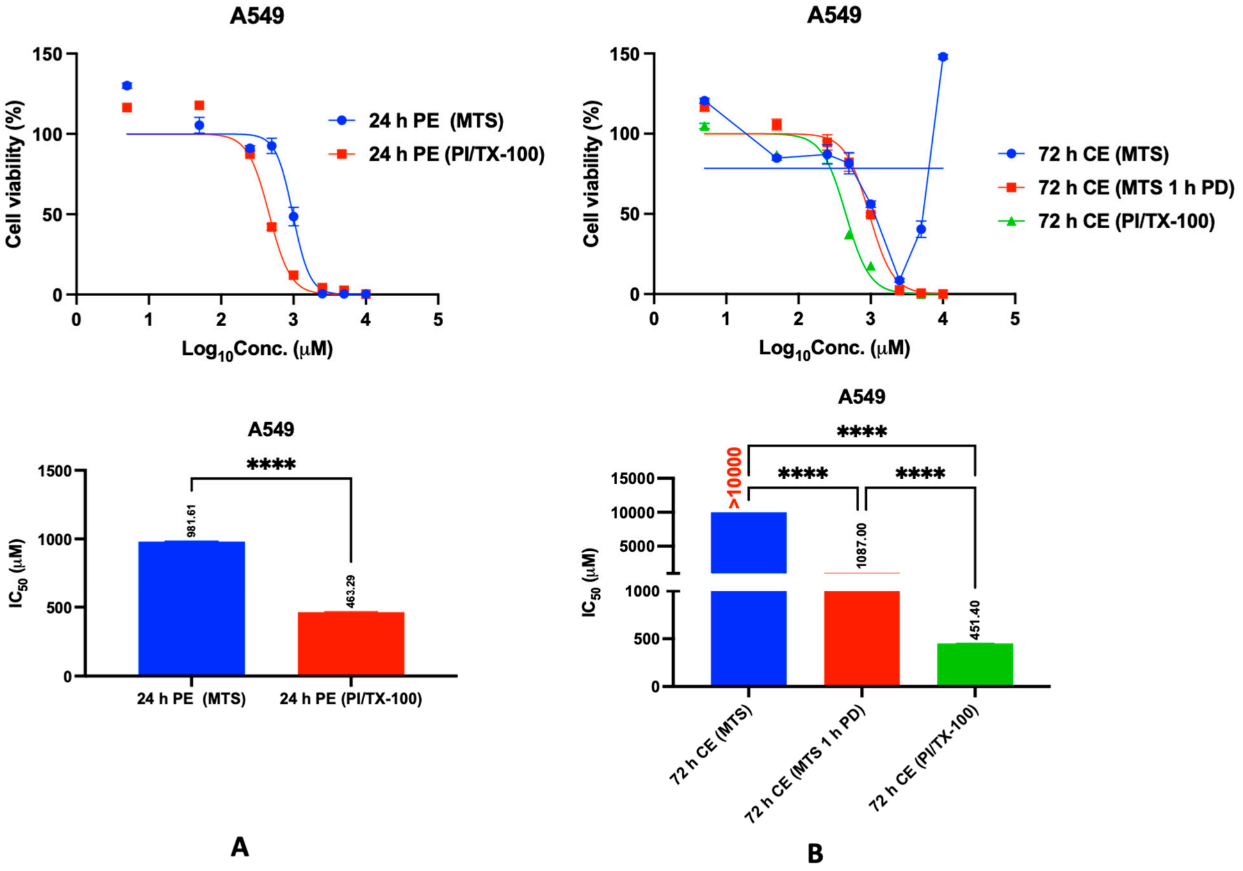

Figure S1. Exposure dependency impact of AA on A549 cells with respect to different viability (colorimetric and fluorometric) assays. Cells were seeded for 24 h, treated with fresh medium containing various concentrations of AA (5 – 10000 μM) and incubated for 24 h (pre-exposure condition: PE) or 72 h (continuous exposure condition: CE). A: For 24-h PE exposure, the cells were treated for 24 h, replaced with fresh medium devoid of AA, and subsequently incubated for 48 h. B: For 72 CE, cells were treated with AA for 72 h. 72 h CE condition was further modified into post-drainage: PD), in which case, after 72 h, the spent growth medium was aspirated and replaced with fresh medium devoid of ascorbic acid 1-2 h before the termination of the experiment. Viability was assessed by MTS or PI/TX-100. IC_{50} values were calculated by non-linear regression (log [Conc. of drug] versus response (% (T-B/C-B)) using the GraphPad Prism analysis software.

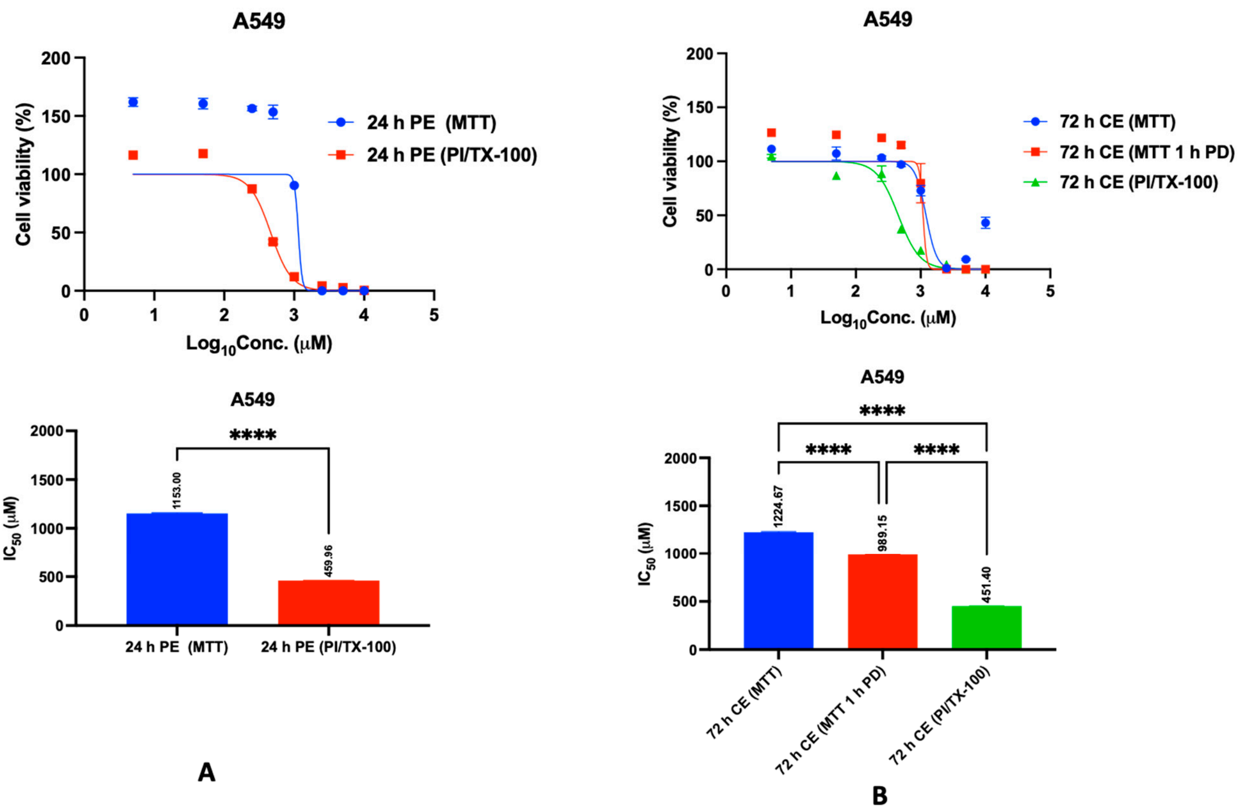

Figure S2. Exposure dependency impact of AA on A549 cells with respect to different viability (colorimetric and fluorometric) assays. Cells were seeded for 24 h, treated with fresh medium containing various concentrations of AA (5 – 10000 μM) and incubated for 24 h (pre-exposure condition: PE) or 72 h (continuous exposure condition: CE). A: For 24-h PE exposure, the cells were treated for 24 h, replaced with fresh medium devoid of AA, and incubated for 48 h. B: For 72 CE, cells were treated with AA for 72 h. 72 h CE condition was further modified into post-drainage: PD), in which case, after 72 h, the spent growth medium was aspirated and replaced with fresh medium devoid of ascorbic acid 1-2 h before the termination of the experiment. Viability was assessed by MTT or PI/TX-100. IC_{50} values were calculated by non-linear regression (log [Conc. of drug] versus response (% (T-B/C-B)) using the GraphPad Prism analysis software.

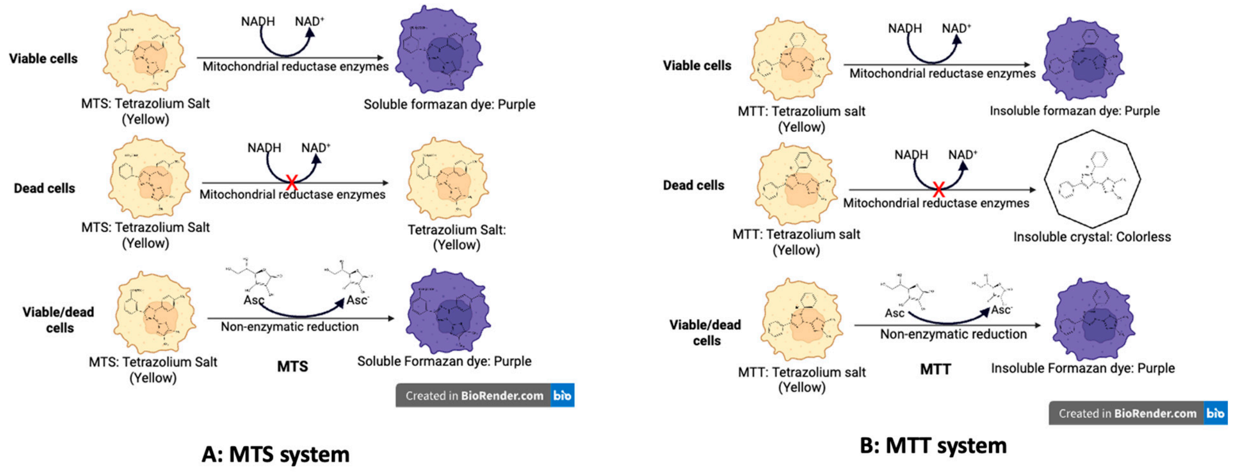

Figure S3. AA enhances formazan formation in the presence of MTS/MTT reagent in cell cultures. In viable cells, NADH reacts with tetrazolium salts of MTS/MTT to form formazan (purple coloration), which indicates the cell's metabolic activity. However, in dead cells, tetrazolium salt of MTS/MTS is not reduced to formazan (yellow coloration is retained). However, in the presence of

ascorbic acid (AA), the formation of formazan (purple coloration) is observed in both live/dead cells; thus, leading to a false positive result. This is because AA can mimic NADH, reducing the tetrazolium salt of MTS to formazan. *Created with Bionder.com.*

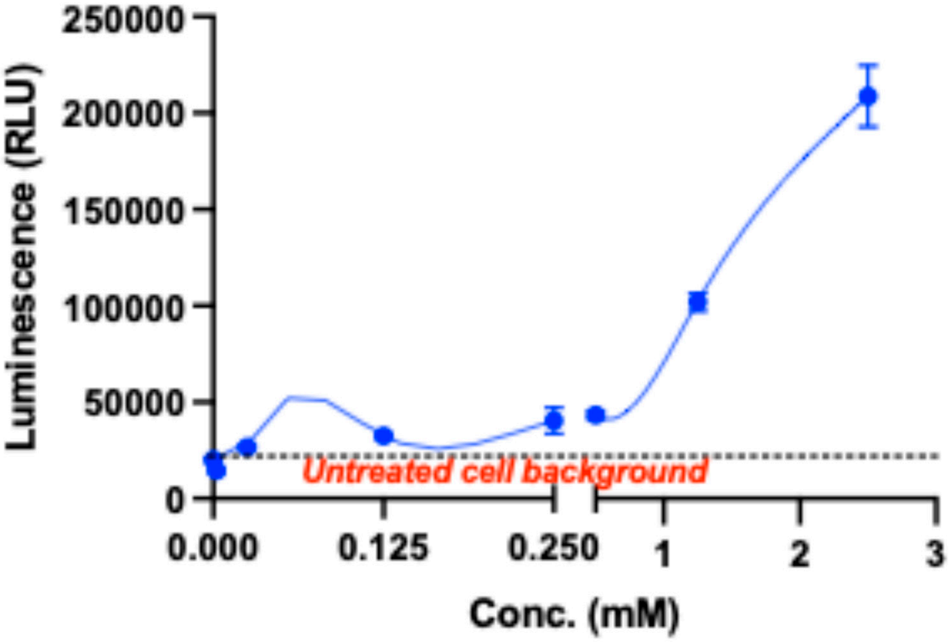

Figure S4. Effect of AA on extracellular ATP levels. MDA-MB-453 (1 x 10^4) cells were incubated for 24 h and treated with various concentrations of AA and 4X RealTime-Glo™ Extracellular ATP Assay Reagent (GA501A), and incubated for 24 h. Luminescence measurements were collected using an iTecan plate reader set at 37 °C.

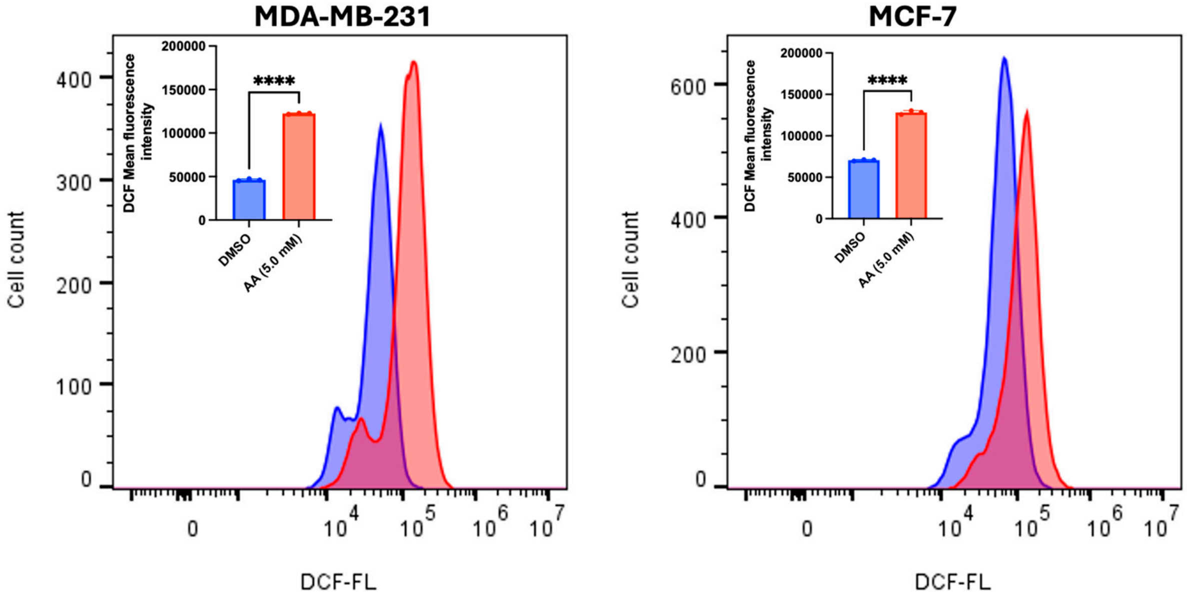

Figure S5. AA mediates ROS generation. MCF-7 and MDA-MB-231 (1 x 10^6) cells were seeded for 24 h and treated with AA (5.0 mM), or DMSO (1%). H2DCF-DA probe (10 µM) was added during treatment and incubated for 1 h. DCF fluorescence was detected by FACS, and mean DCF fluorescence intensities of triplicate samples were compiled.

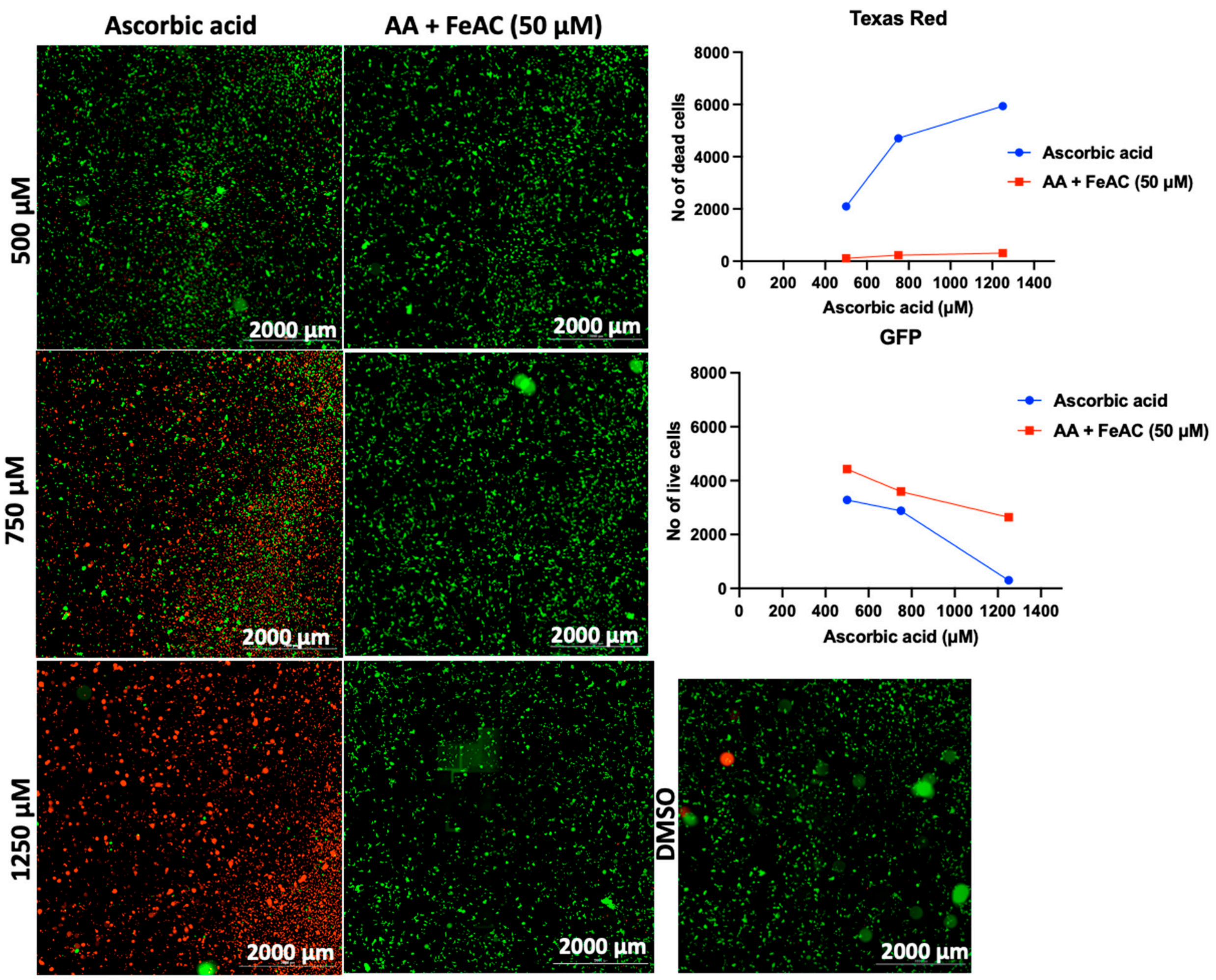

Figure S6. Effect of exogenous iron (FeAC) on the antiproliferative activity of ascorbic acid (AA) in LNCaP cells. A: Cells were seeded for 24 h, treated with various concentrations of AA. For combination therapy, the cells were co-treated with various concentrations of AA at a fixed concentration of FeAC (50 μM) and subsequently incubated for 24 h. Cells were stained with fluorescein diacetate (FDA) and propidium iodide, and imaged at the threshold (GFP: 469 and 525 nm, Texas Read: 586 and 647 nm).

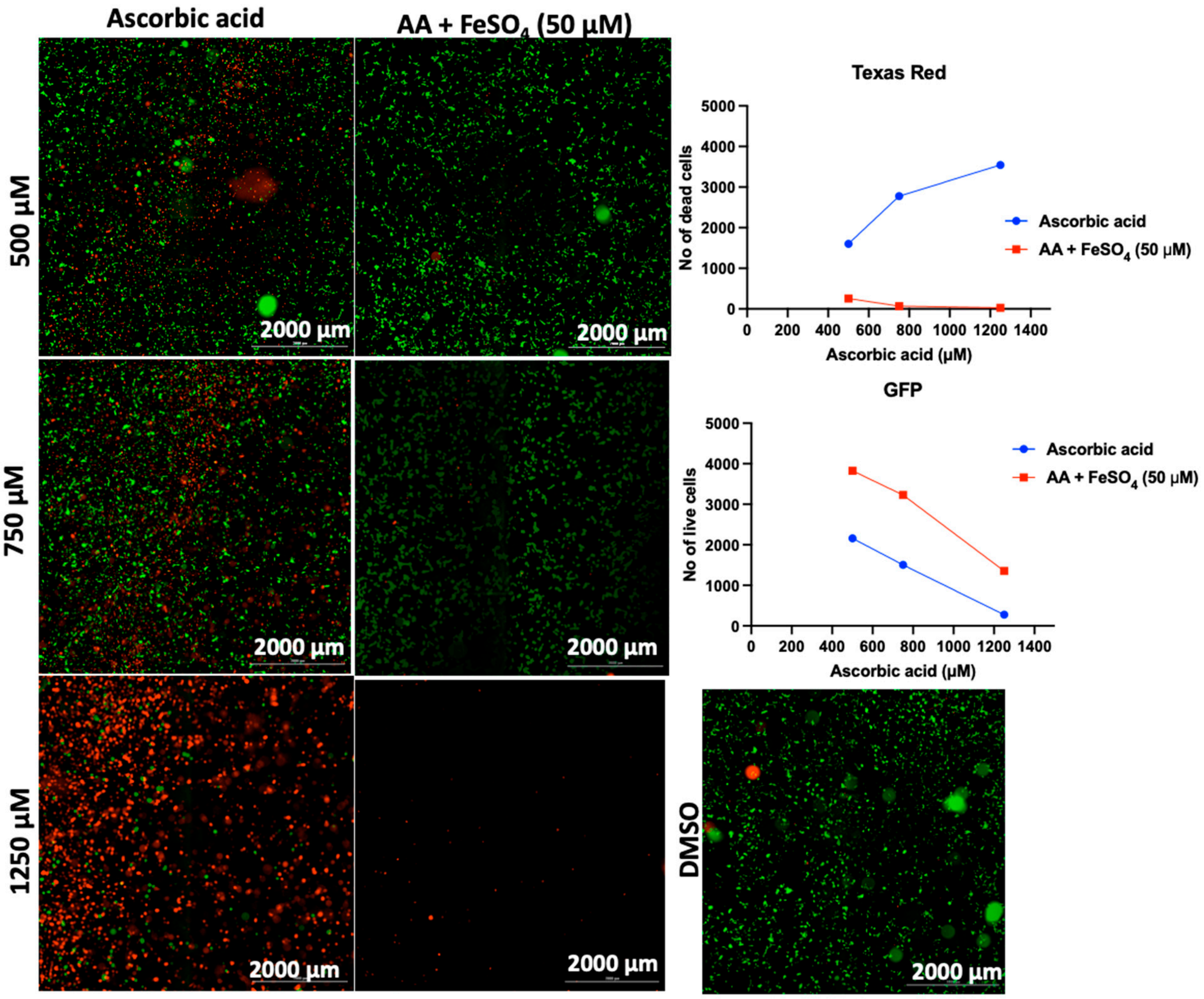

Figure S7. Effect of exogenous iron ($FeSO_4$) on the antiproliferative activity of ascorbic acid (AA) in LNCaP cells. A: Cells were seeded for 24 h, treated with various concentrations of AA. For combination therapy, the cells were co-treated with various concentrations of AA at a fixed concentration of $FeSO_4$ (50 μM) and subsequently incubated for 24 h. Cells were stained with fluorescein diacetate (FDA) and propidium iodide, and imaged at the threshold (GFP: 469 and 525 nm, Texas Read: 586 and 647 nm).

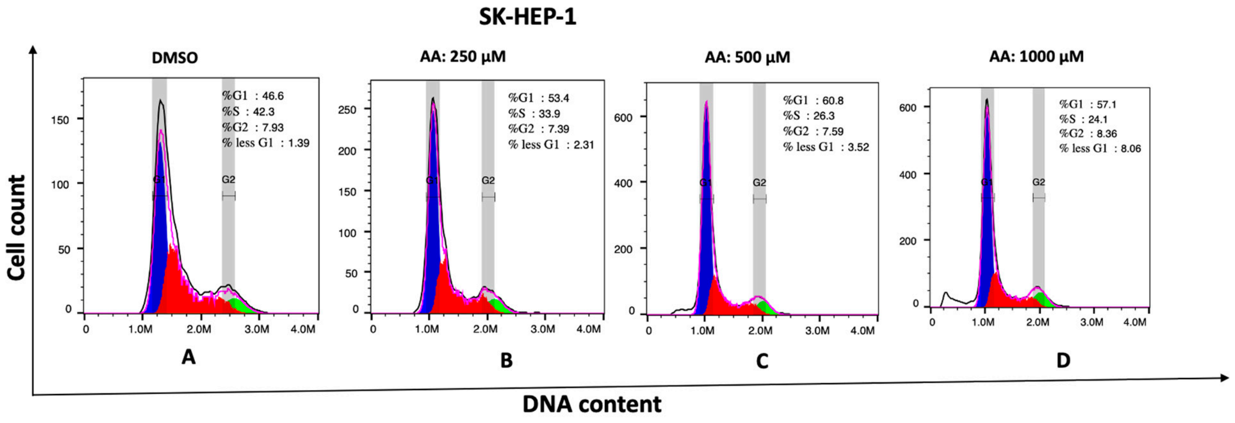

Figure S8. Cell cycle analysis of SK-HEP-1 cells. (A-D) Cell cycle analysis by flow cytometry of SK-HEP-1 cells treated with DMSO (A: 1%) or ascorbic acid (B: 250, C: 500, and D: 1000 μM). <G1 cells represent apoptotic cells.

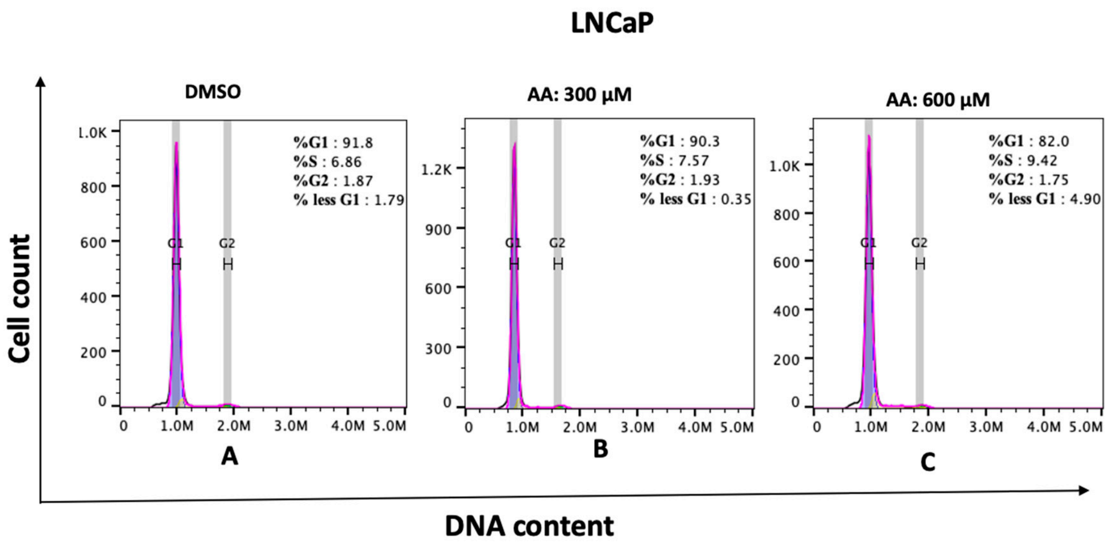

Figure S9. Cell cycle analysis of LNCaP cells. (A-C) Cell cycle analysis by flow cytometry of LNCaP cells treated with DMSO (A: 1%) or ascorbic acid (B: 300, and C: 600 μM). <G1 cells represent apoptotic cells.

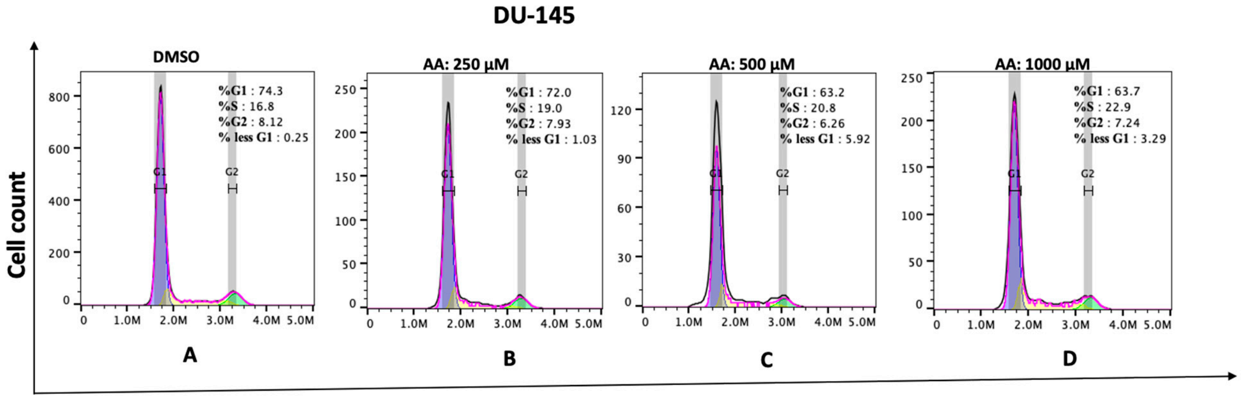

Figure S10. Cell cycle analysis of DU-145 cells. (A-D) Cell cycle analysis by flow cytometry of DU-145 cells treated with DMSO (A: 1%) or ascorbic acid (B: 250, C: 500, and D: 1000 μM). <G1 cells represent apoptotic cells.

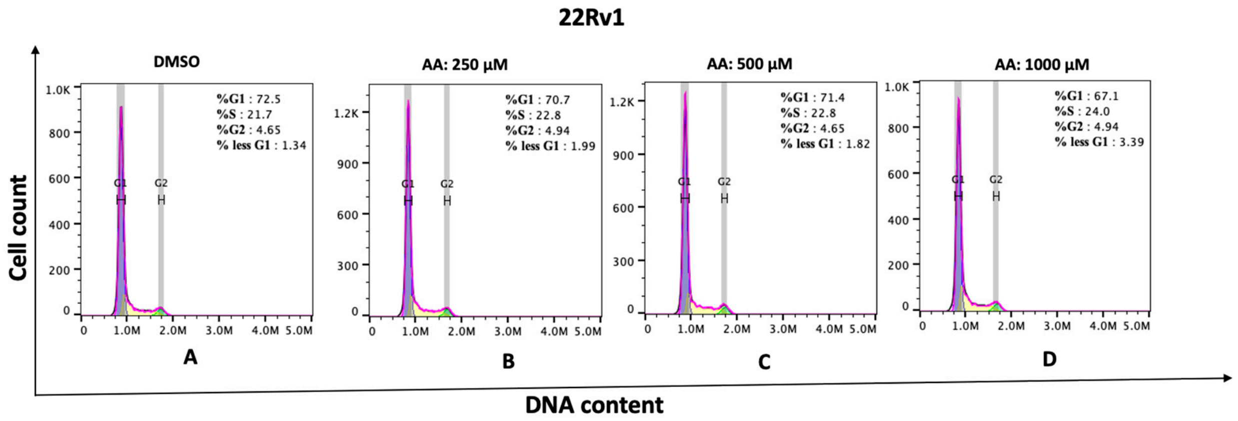

Figure S11. Cell cycle analysis of 22Rv1 cells. (A-D) Cell cycle analysis by flow cytometry of 22Rv1 cells treated with DMSO (A: 1%) or ascorbic acid (B: 250, C: 500, and D: 1000 μM). <G1 cells represent apoptotic cells.

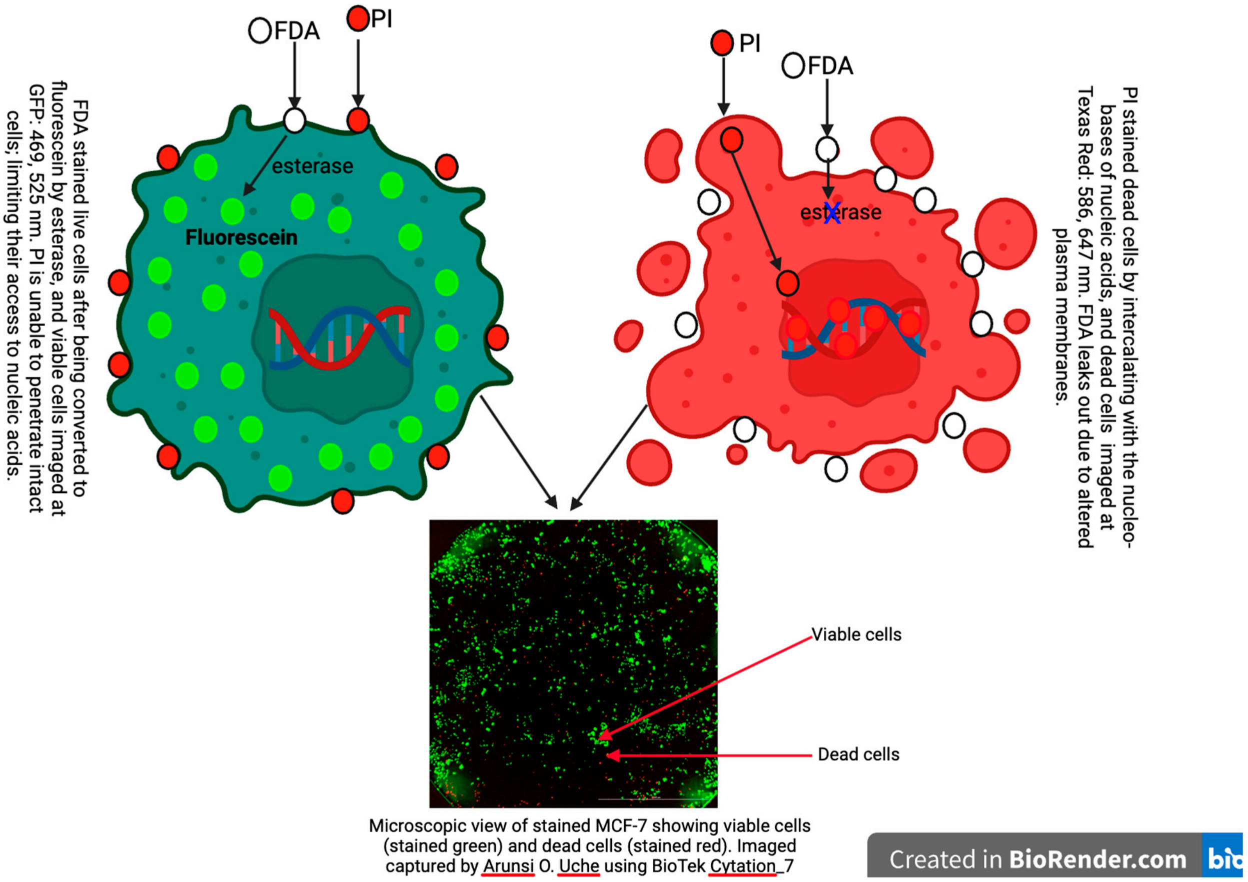

Figure S12. Reaction mechanisms of FDA/PI viability assays. In live cells, esterase converts FDA to fluorescein, which produces fluorescence that can be detected with GFP filter (469, 525 nm). However, in dead cells, FDA remained untransformed due to low activity of esterase. In dead cells, PI enters the nucleus, intercalates with DNA and produces fluorescence which can be detected with Texas Red filter (586 and 647 nm).

Table S1. Comparison of the anticancer activity of AA at pre-exposure and constant exposure conditions.

Cell line	IC_{50} (µM)			
	MTS		PI/TX-100	
	24-h	72-h	24-h	72-h
LNCaP	685.60 ± 5.30	>10000	540.90 ± 8.83	317.60 ± 8.49
DU-145	871.00 ± 4.47	>10000	690.00 ± 7.20	413.20 ± 1.49
MCF-7	1256.00 ± 7.80	>10000	1113.00 ± 9.20	728.70 ± 6.47
MDA-MB-231	3895.00 ± 10.80	>10000	2823.00 ± 6.50	1650.00 ± 5.53
HepG2	850.20 ± 4.66	>10000	753.50 ± 11.85	656.20 ± 14.61
A549	1109.80 ± 12.34	>10000	515.30 ± 6.30	445.20 ± 5.35

Table S2. Determination of the anticancer activity of AA by MTS cell viability assay.

Cell lines	MTS	Type of Cancers
LNCaP	>10000	Prostate cancer
DU-145	>10000	Prostate cancer
C4-2B	>10000	Prostate cancer
22Rv1	>10000	Prostate cancer
MDA-MB-453	>10000	Breast cancer
MDA-MB-231	>10000	Breast cancer
MCF-7	>10000	Breast cancer
SK-HEP-1	>10000	Liver Cancer
HepG2	>10000	Liver Cancer
Huh7	>10000	Liver Cancer
A549	>10000	Lung cancer
RWPE1	>10000	Normal cell
Vero	>10000	Normal cell
